# 5-Fluorouracil modulates motility and biofilm-associated gene expression in *Pseudomonas aeruginosa*

**DOI:** 10.1371/journal.pone.0354473

**Published:** 2026-07-23

**Authors:** Amani A. Niazy, May M. Alrashed, Rhodanne Nicole A. Lambarte, Terrence S. Sumague, Abdurahman A. Niazy

**Affiliations:** 1 Department of Clinical Laboratory Sciences, College of Applied Medical Sciences, King Saud University, Riyadh, Saudi Arabia; 2 Molecular and Cell Biology Laboratory, Prince Naif bin Abdulaziz Health Research Center, College of Dentistry, King Saud University Medical City, King Saud University, Riyadh, Saudi Arabia; 3 Department of Oral Medicine and Diagnostic Sciences, College of Dentistry, King Saud University, Riyadh, Saudi Arabia; Islamic Azad University, IRAN, ISLAMIC REPUBLIC OF

## Abstract

*Pseudomonas aeruginosa* is an opportunistic pathogen in which motility, biofilm formation, and stress adaptation contribute to virulence. Drug repurposing represents a practical strategy for identifying compounds that influence these processes. In this study, the effects of 5-fluorouracil (5-FU) on motility, extracellular DNA (eDNA) production, and gene expression were examined in *P. aeruginosa* PAO1. Swimming, swarming, and twitching motility assays were performed in the presence of increasing concentrations of 5-FU. Transcriptional responses of motility, rhamnolipid, and DNA repair-associated genes were evaluated using quantitative real-time PCR. eDNA levels were quantified using fluorescence-based assays and visualized by confocal laser scanning microscopy. Exposure to 5-FU resulted in concentration-dependent reductions in swimming, swarming, and twitching motility. These changes were associated with downregulation of multiple genes involved in flagellar and type IV pili function, including *motA*, *flhA*, *fliD*, *pilA*, and *pilI*, and reduced expression of *lasB* and *rhlAB*. At 24 h, eDNA levels were decreased relative to untreated controls, whereas at 48 h, higher concentrations of 5-FU were associated with increased eDNA accumulation and upregulation of several DNA repair genes, including *xthA*, *nth*, *recJ*, *sbcB*, and *eddB*. These results indicate the multifaceted effects of 5-FU on important virulence factors of *P. aeruginosa.*

## Introduction

Healthcare-associated infections are a growing global challenge that strain healthcare systems and worsen patient outcomes. Among opportunistic pathogens, *Pseudomonas aeruginosa* is notable for its environmental adaptability, metabolic versatility, and ability to persist in hospital reservoirs while causing disease in both immunocompromised and immunocompetent hosts [1,2]. Its success as a pathogen is largely attributed to a broad spectrum of virulence factors that facilitate adhesion, colonization, immune evasion, biofilm formation and tissue damage [3]. This threat is further exacerbated by the intrinsic resistance of *P. aeruginosa* to multiple antibiotics and its extraordinary ability to acquire additional resistance mechanisms, which limit treatment options [4,5]. Consequently, both the World Health Organization and the Centers for Disease Control and Prevention have listed *P. aeruginosa* as a priority pathogen requiring urgent development of new therapeutic strategies [6,7].

Motility plays a critical role during the early stages of *P. aeruginosa* infection by facilitating surface exploration and initial attachment, which precede stable adhesion and subsequent biofilm development on host tissues and implanted medical devices [8,9]. Accordingly, impairing motility has emerged as an attractive antivirulence strategy aimed at disrupting bacterial coordination rather than bacterial viability [8,10,11]. In parallel, extracellular DNA (eDNA) is a critical structural component of *P. aeruginosa* biofilms, contributing to matrix cohesion, mechanical stability, and persistence, while also reflecting physiological stress and adaptive responses during biofilm maturation [12,13]. Current evidence indicates that eDNA originates from multiple sources including the membrane vesicles released by viable cells and autolysis of a subpopulation that contributes matrix material to support community integrity [14]. Because motility, rhamnolipid production, and eDNA release contribute to early biofilm development and are regulated by shared stress and virulence pathways, disruption of these processes represents a rational antivirulence-oriented strategy for impairing the ability of the organism to cause infection without exerting strong bactericidal pressure [3,15].

As antimicrobial resistance intensifies, drug repurposing has emerged as an efficient strategy for identifying new antibacterial agents by leveraging existing pharmacological and safety data to reduce development time, cost, and risk [16–19]. Among anticancer compounds, 5-fluorouracil (5-FU) has drawn attention for its antibacterial and antivirulence activity [20,21]. Related fluoropyrimidine and small-molecule studies have also reported inhibition of virulence-associated functions in *P. aeruginosa* [22–25]. Furthermore, synergistic effects between 5-FU and conventional antibiotics such as gentamicin have been reported suggesting its potential as an adjuvant therapy [26,27]. However, the precise molecular mechanisms underlying its antibiofilm and antivirulence effects remain poorly understood.

This study aimed to elucidate the effects of 5-FU on biofilm-associated phenotypic and transcriptional traits in *P. aeruginosa* PAO1. Specifically, we investigated its impact on bacterial motility using phenotypic and gene expression analyses. We then evaluated its influence on rhamnolipid gene expression and eDNA production, and explored the transcriptional response of DNA repair genes. Additionally, molecular docking was performed to provide complementary and structure-informed hypotheses regarding potential drug-protein interactions that may underlie observed transcriptional and phenotypic changes. By integrating these findings, we sought to provide a comprehensive understanding of how 5-FU affects *P. aeruginosa* virulence-related pathways, thus contributing to the broader goal of identifying novel therapeutic strategies against resilient *P. aeruginosa* infections.

## Materials and methods

### Bacterial strain, drug, and growth media

All experiments were performed using *Pseudomonas aeruginosa* PAO1 kindly provided by Dr. Lee Hughes (University of North Texas, Denton, USA). The strain was maintained at −80 °C in tryptic soy broth containing 10% glycerol. For culture revival, the stock was streaked onto tryptic soy agar (TSA) and incubated aerobically at 37 °C for 18–20 h. A single, well-isolated colony was then transferred into 10 mL of *Pseudomonas* minimal medium (PsMM) following the previously described method [28]. The bacterial culture was used for subsequent experiments once it reached the mid-logarithmic growth phase corresponding to an optical density at 600 nm (OD₆₀₀) of 0.5–0.6, as measured with a spectrophotometer (Libra S22, Biochrom Ltd., Cambridge, UK).

5-FU powder (Ebewe Pharma, FAREVA Unterach GmbH, Austria) was reconstituted in sterile saline (Pharmaceutical Solutions Industry, Saudi Arabia) to prepare a 5 mg/mL stock solution following the manufacturer’s instructions. The resulting suspension remained stable at −5 °C for up to 28 days [29].

### Motility assays

#### Preparation of standardized bacterial inoculum.

To ensure consistency across all motility assays, the bacterial inoculum was standardized for every plate. The primary objective was to normalize the initial bacterial load in each motility medium so that any observed differences reflected the true effect of the drug rather than variations in inoculum density. This approach minimized experimental bias attributable to inoculum variability. The procedure was performed according to published protocols [30–32] with modifications. Briefly, 1 mL of the mid-logarithmic-phase culture of PAO1 was transferred into a microcentrifuge tube and centrifuged at 10,000 rpm for 2 min. The supernatant was discarded, and the bacterial pellet was resuspended in 50 µL of PsMM. A 5 µL aliquot of this suspension was used to inoculate the motility media according to the specific assay procedures.

### Incorporation of drug into motility media

For all motility assays, 5-FU was incorporated into the motility media at final concentrations of 0.1, 0.5, 12, and 100 µg/mL prior to solidification. Following autoclaving, the molten medium was divided into five sterile flasks corresponding to the untreated control and the four different 5-FU concentrations. The drug was added to each flask once the medium had cooled to approximately 50–55 °C, just before solidification, to ensure uniform distribution without affecting agar integrity. After thorough mixing, the media were poured into sterile Petri dishes and allowed to solidify at room temperature without refrigeration. Once solidified, PAO1 was inoculated according to the specific protocol for each motility assay [30–32].

### Swarming motility assay

Swarming motility was evaluated on semi-solid Luria broth medium containing 0.5% agar and 0.5% glucose. A 5 µL aliquot of the previously prepared PAO1 suspension was inoculated onto the surface of the agar. Plates were incubated aerobically at 37 °C for 20–23 h without inversion, and the swarming zone was measured across the diameter of the motility area [30].

### Swimming motility assay

The swimming agar plates contained 1.0% tryptone, 0.5% sodium chloride, and 0.3% agar. After incorporation of the drug and solidification of media, 5 µL of the prepared bacterial suspension was inoculated onto the surface of the medium. Plates were then incubated without inversion at 30 °C for 20–23 h. Swimming motility was evaluated by measuring the diameter of the motility zone [31].

### Twitching motility assay

Twitching motility was assessed on Luria broth medium (MOLEQULE-ON, Auckland, New Zealand) solidified with 1.5% agar. After incorporation of the drug and solidification of media, 5 µL of the prepared bacterial suspension was inoculated beneath the agar surface at a 45° angle. Plates were incubated aerobically at 37 °C for 20–23 h. Following incubation, the agar layer was carefully removed, and the plates were stained with 2% crystal violet for 20 min at room temperature for visualization. The twitching zone was measured across the diameter of the motility area [32,33].

### Quantitative real-time PCR (qPCR)

Six-well culture plates (Greiner bio-One, Frickenhausen, Germany) containing standardized PAO1 cultures as described above, and four concentrations of 5-FU (0.1, 0.5, 12, 100 µg/mL) were incubated for 24 or 48 h at 37 °C in static conditions. After incubation, the wells were gently washed with sterile phosphate-buffered saline (PBS) to remove planktonic cells, and the remaining biofilm cells were scraped and resuspended in PsMM. Next, 1 mL of the scraped biofilm cells was centrifuged at 10,000 rpm for 5 min, and total RNA was extracted using the Quick-RNA fungal/bacterial miniprep kit (Zymo Research, cat# R2014, Irvine, CA, USA) according to the manufacturer’s instructions with minor modifications. RNA concentration and purity were verified using a BioSpectrometer® basic (Eppendorf, Germany).

Quantitative real-time PCR was used to assess the expression of biofilm- and motility-related genes in PAO1; the genes used in expression analysis are listed in Table 1. For both 24 and 48 h biofilms, three independent biological replicates were performed, with each biological replicate analyzed in technical triplicates. Extracted RNA samples were stored at −80 °C until cDNA synthesis and qPCR analysis. Total RNA samples were reverse-transcribed into cDNA using the Haven Scientific RT Ace First-Strand cDNA synthesis kit (KAUST, Thuwal, Saudi Arabia) according to the manufacturer’s protocol. qPCR was subsequently performed with Haven Scientific EverGreen Universal Real-Time PCR master mix (KAUST, Thuwal, Saudi Arabia) on an ABI 7500 Real-time PCR system (Applied Biosystems, USA) under the following cycling conditions: 94 °C for 12 min; 40 cycles of 95 °C for 15 s, 65 °C for 30 s, and 72 °C for 30 s. The expression levels of the target genes were measured relative to the untreated control and normalized to the expression of the endogenous reference gene (16S ribosomal RNA gene). Relative expression levels were calculated using the 2^−ΔΔCt^ method [43].

**Table 1:** Target genes tested in expression studies.

Gene	Primer Sequence (5’ – 3’)	T_m_ (°C)	Reference
***pilA*** Type IV fimbrial precursor PilA	F: TGCGCGTTCGGAAGGT R: CGACTCTTCAACAGTGGTCTTCA	62.3 61.6	[34]
***pilI*** Twitching motility protein PilI	F: GCACTGCAACCCTTCATTCAT R: CGCATGCGGGCTGAAC	59.9 62.2	[34]
***pilS*** Two-component sensor PilS	F: ACCTGGTCATCGAGAACGTC R: GCTCAGCACCTGGTTCAACT	62.1 62.3	[35]
***fliC*** Flagellin type B	F: CAGTGCCAAGGACGATGCT R: AACGTTCAGACCGCTGATCTG	62.5 61.7	[34]
***fliD*** Flagellar capping protein FliD	F: TGGCTGGCACCTACCAGATC R: GGCCTGGAGGGCAATCTT	65.0 64.1	[34]
***fleQ*** Transcriptional regulator FleQ	F: AGCCAGCATTTGGCCACTA R: ACTACCGCCTCAACGTATTCC	61.1 61.2	[36]
***flhA*** Flagellar biosynthesis protein FlhA	F: CGGACAACAAGCAAGTCACCATCG R: ATCGGCGGCGAAGAAGCGTTTGAC	65.1 67.8	[37]
***motA*** Chemotaxis protein MotA	F: GAGAATCCCGAGGAAGGTG R: TCATGGTGGTGCTGAAGAAA	61.8 59.1	[38]
***lasB*** Elastase B	F: GGAATGAACGAAGCGTTCTC R: GGTCCAGTAGTAGCGGTTGG	58.6 63.0	[39]
***rhlC*** Rhamnosyltransferase 2	F: ATCCATCTCGACGGACTGAC R: GTCCAGGTCGTCGATGAAC	61.8 61.2	[40]
***rhlAB*** Rhamnolipid biosynthesis operon	F: TCAACGAGACCGTCGGCAAATACCT R: AATCCCGTACTTCTCGTGAGCGATG	66.4 64.8	[40]
***nth*** Endonuclease III	F: GTCGGGGTGAACAAGGCTAC R: TCGCCTTGCTGTTGTAGAGG	60.0 57.0	[41]
***xthA*** Exodeoxyribonuclease III	F: CGAATGGCTGGCTACCTTGA R: CGGTAGTCGAACCAGCTGAA	57.0 57.0	[41]
***recJ*** Single-stranded-DNA-specific exonuclease RecJ	F: ACTTTCCCGAGCCGATGTTC R: GCATTCGCTTTTCAGCACCA	57.0 55.0	[41]
***sbcB*** Exodeoxyribonuclease I	F: AAGCAGATCCAGGTCAACCG R: AACAGCTCGGCTTTTTGCTG	57.0 55.0	[41]
***eddB*** Extracellular DNA degradation protein, EddB	F: CCAGCTTCAACGTGCTCAAC R: TTCTGCCGTTGGAACTCCTC	57.0 57.0	[41]
**16S rRNA**	F: GCGCAACCCTTGTCCTTAGTT R: TCTCACCGGCAGTCTCCTTAG	62.2 60.9	[42]

Abbreviations: F, forward primer; R, reverse primer; Tm, melting temperature.

### Extracellular DNA (eDNA) Quantification

Biofilms of PAO1 were cultivated in six-well plates (Greiner bio-One, Frickenhausen, Germany) containing PAO1 suspensions and 5-FU at final concentrations of 0.1, 0.5, 12, 100 µg/mL and incubated at 37 °C for 24 or 48 h under static conditions. Following incubation, eDNA was measured following the protocol of Zatorska et al. with some modifications [44]. Briefly, planktonic cells were gently removed by washing with sterile PBS buffer. The remaining biofilms were stained with 1 µM TOTO®-1 iodide (Thermo Fisher, Waltham, MA, USA) for 20 min in the dark at room temperature. The stained biofilm was then carefully scraped from the well surface and transferred to a black 96-well microplate. Fluorescence intensity was measured at excitation/emission wavelengths of 514/531 nm using a microplate reader system from BioTek Instruments (Winooski, VT, USA) and fluorescence intensity was expressed relative to the untreated control.

### Confocal laser scanning microscopy (CLSM) of eDNA

PAO1 biofilms treated with different concentrations of 5-FU were cultured on coverslips in six-well culture plates at 37 °C for 24 or 48 h. After incubation, the broth was removed, and the biofilms were gently washed three times with PBS to remove planktonic cells. The coverslips were carefully transferred to a new six-well plate, and 200 µL of 2 µM TOTO®-1 iodide (Thermo Fisher, Waltham, MA, USA) was added onto each coverslip, followed by incubation for 20 min in the dark at room temperature according to the manufacturer’s instructions. The PAO1 biofilms were imaged using a Nikon C2 confocal laser scanning microscope (Nikon Instruments Inc., Tokyo, Japan) using 488 nm/ < 550 for TOTO®-1 iodide. The representative images were captured using a 20 × air objective lens (0.75 NA) and NIS-Elements Advanced Research Software (version 4.0, Nikon, Japan).

### Protein-protein interaction (PPI) network analysis

The list of PAO1 genes was input in the Search Tool for the Retrieval of Interacting Genes database (STRING; version 12.0) [45] through the web interface; we specified the organism using “*Pseudomonas aeruginosa* PAO1”. Gene names were mapped according to STRING database annotation; due to organization and regulatory relationships, *rhlAB* was represented by its transcriptional regulator *rhlR.* The protein-protein interaction network was constructed using the default minimum interaction score of 0.4 and without additional interactors. The generated PPI network was further analyzed with Cytoscape (version 3.10.3) [46] using cytoHubba (version 0.1). The Maximal Clique Centrality (MCC) algorithm identified the key nodes in the network. Edge betweenness centrality analysis was performed using NetworkAnalyzer (version 4.5.0) to identify key node-to-node connections within the network. The node color, size, and shape were visualized by mapping the gene regulation, fold change, and node rank, respectively; network edge thickness values were drawn according to the EdgeBetweenness score.

### Molecular docking analysis

The protein crystal structures of transcriptional regulator FleQ (fleQ) (Protein Data Bank (PDB) ID: 5EXP), flagellin type B (fliC) (PDB ID: 8erm), flagellar capping protein FliD (fliD) (PDB ID: 5fhy), elastase LasB (lasB) (PDB ID: 8r1b), and type IV fimbrial precursor PilA (pilA) (PDB ID: 8v7p) were obtained from the Protein Data Bank [47]. The protein three-dimensional (3D) structure of extracellular DNA degradation protein (eddB), flagellar biosynthesis protein FlhA (flhA), chemotaxis protein MotA (motA), twitching motility protein PilI (pilI), two-component sensor PilS (pilS), rhamnosyltransferase 2 (rhlC), endonuclease III (nth), exodeoxyribonuclease I (sbcB), exodeoxyribonuclease III (xthA), and single-stranded-DNA-specific exonuclease Rec (recJ) were predicted using Iterative Threading Assembly Refinement (I-TASSER) [48] based on their amino acid sequences obtained from the *Pseudomonas* Genome Database [49]. PAO1 protein locus tags used in this study are outlined in S1 Table. Protein structures were prepared using the Protein Repair and Analysis Server (PRAS) and AutoDock tool protocol [50,51]. The 3D structure of 5-FU was downloaded from the PubChem database (PubChem ID: 3385) [52]. The ligand was optimized using Avogadro software (version 1.2.0) [53] and then exported to mol2 format. All PDBQT files for VINA docking for receptor proteins and ligands were generated using AutoDock Tools. The grid box dimensions, center coordinates, and VINA parameters are outlined in S2 Table. AutoDock Vina software (version 1.2.7) [54] was used to perform molecular docking, and the best docking scores were selected. The output complex poses were visualized using PyMol software (version 3.0.3). Discovery Studio 2024 Client (Dassault Systemes BIOVIA, version 24.1.0) [55] was used to visualize 3D protein-ligand interactions and to identify different non-covalent interactions. ChimeraX software (version 1.7.1) was used to generate the protein-ligand complex 3D conformations.

### Statistical analysis

All data are presented as mean ± standard deviation (SD). Values were derived from the average of three independent biological replicates, with each condition measured in technical triplicate. Statistical differences were assessed using one-way analysis of variance (ANOVA) based on the investigated variables followed by either Dunnett’s or Bonferroni’s post hoc analysis test to evaluate differences across the treatment groups. Statistical analyses were conducted utilizing GraphPad Prism (version 10.6.1 (799), La Jolla, CA, USA; https://www.graphpad.com). A p-value of < 0.05 was deemed statistically significant.

## Results

### Motility

**Fig 1:**
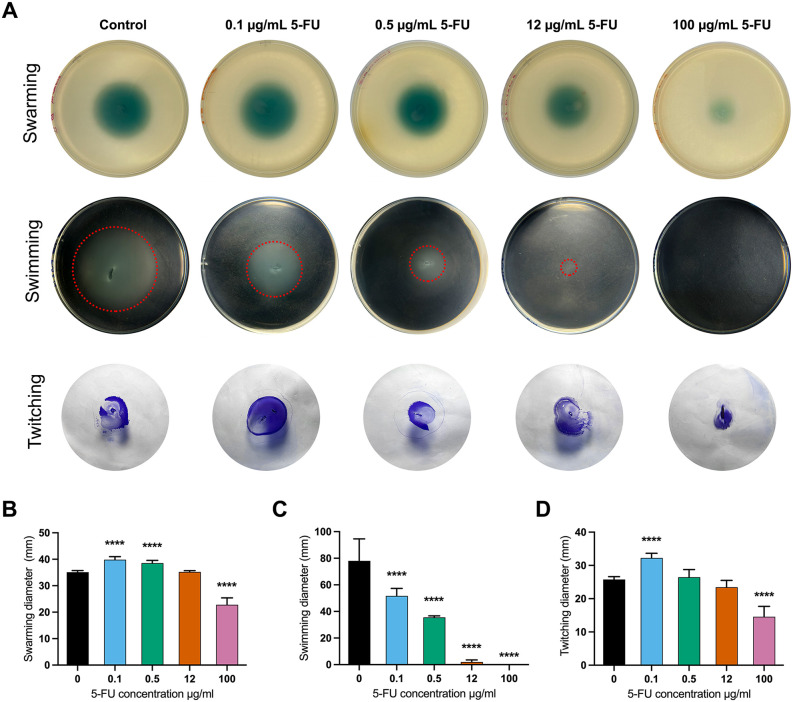
Effect of 5-FU at concentrations of 0.1, 0.5, 12, and 100 µg/mL on the motility of PAO1. The figure shows representative images for swarming, swimming and twitching motility, and the graph shows measurements of motility zone diameter (mm) of the treated sample versus untreated control (n = 3). Asterisks indicate levels of statistical significance: p < 0.0001 (****). Data represent the mean ± SD of three independent experiments.

Swarming motility was significantly affected by 5-FU in a concentration-dependent pattern. The mean swarming diameter in the control was 35.11 ± 0.60 mm with a significant increase at 0.1 µg/mL (39.83 ± 1.17 mm) and 0.5 µg/mL (38.56 ± 1.01 mm) followed by a gradual reduction to 35.22 ± 0.44 mm at 12 µg/mL and 22.78 ± 2.64 mm at 100 µg/mL (Fig 1B).

Similarly, swimming motility of PAO1 was influenced by 5-FU. The mean swimming diameter in the untreated control was 78 ± 16.6 mm whereas exposure to 0.1, 0.5, and 12 µg/mL of 5-FU significantly reduced motility to 51 ± 5.7 mm, 35.6 ± 1.1 mm, and 2 ± 1.6 mm, respectively. At 100 µg/mL, no detectable swimming zone or visible bacterial growth was observed on the agar surface (Fig 1A and 1C).

Twitching motility was also affected in a concentration-dependent manner. The mean twitching zone diameter of the control (25.78 ± 0.83 mm) showed a significant increase at 0.1 µg/mL (32.25 ± 1.39 mm) but decreased progressively at higher concentrations reaching 26.44 ± 2.30 mm at 0.5 µg/mL, 23.44 ± 2.07 mm at 12 µg/mL, and 14.56 ± 3.13 mm at 100 µg/mL (Fig 1D).

### eDNA

**Fig 2:**
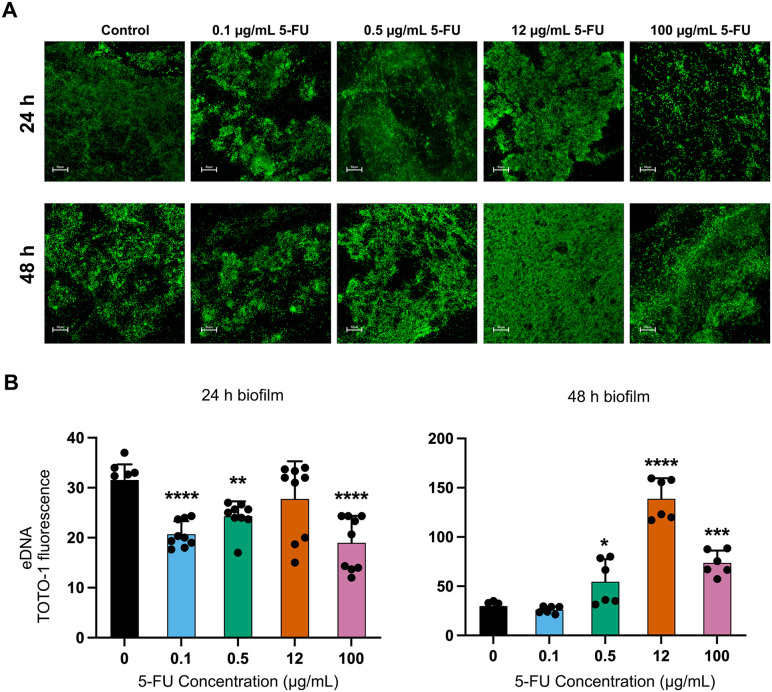
Effect of 5-FU on extracellular DNA (eDNA) content in PAO1 biofilms. (A) Representative CLSM images of PAO1 biofilms after treatment for 24 and 48 h with 5-FU. Biofilms of untreated control and treatment with 0.1, 0.5, 12.0, and 100 µg/mL 5-FU. Scale bar: 50 µm. (B) eDNA fluorescence intensity in 24 and 48 h PAO1 biofilms treated with increasing concentrations of 5-FU. Fluorescence was measured at Ex/Em = 514/531 nm using TOTO®-1 iodide staining. Data represent mean ± SD from three independent experiments. Asterisks indicate levels of statistical significance: *p* < 0.05 (*), p *<* 0.01 (**), *p* < 0.001 (***), and *p* < 0.0001 (****).

Quantification of eDNA revealed a concentration- and time-dependent response to 5-FU treatment. After 24 h, eDNA levels showed a significant reduction at all tested concentrations versus the untreated control. By 48 h, however, low concentrations of 5-FU (0.1 and 0.5 µg/mL) maintained reduced eDNA levels, while treatment with 12 and 100 µg/mL resulted in a significant increase in eDNA fluorescence intensity (Fig 2).

### Gene expression analysis

**Fig 3:**
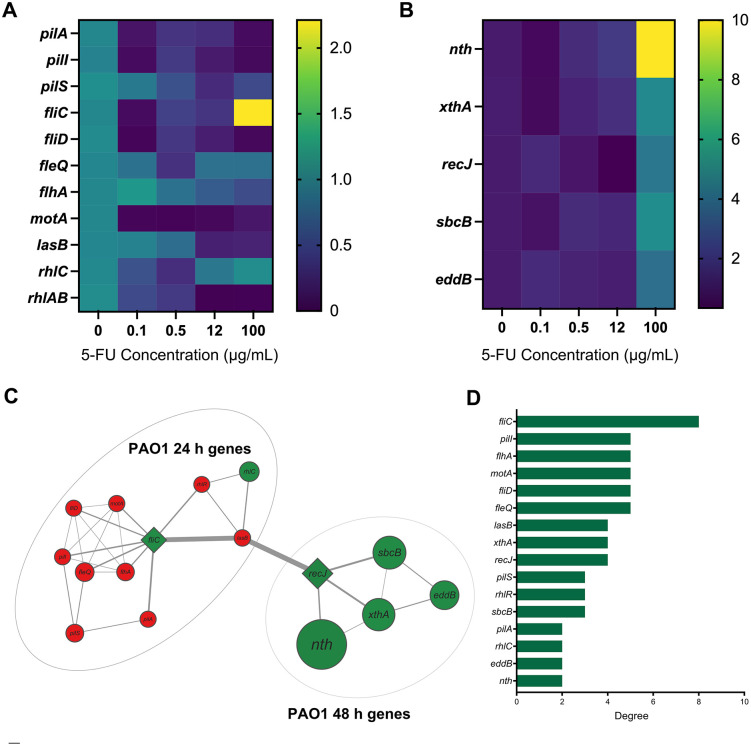
Integrated transcriptional, protein–protein interaction (PPI), and network topology of PAO1 genes following 5-FU exposure. (A) Differential gene expression at 24 and (B) 48 h relative to untreated controls. (C) PPI network constructed from all analyzed genes. Node color indicates transcriptional direction (red, downregulated; green, upregulated); node size reflects the magnitude of the fold-change value. Triangular nodes represent proteins with the highest maximal clique centrality (MCC) scores, thus identifying topologically critical nodes. The edge thickness corresponds to the edge betweenness centrality, thus highlighting interactions that act as major connectors within the network. (D) Degree centrality analysis showing the number of direct interactions per node within the network. Hub genes were identified using topological analyses, including MCC and edge betweenness analyses. MCC was used to identify densely interconnected nodes, where higher scores indicate greater functional importance. Edge betweenness depicts the critical interactions reflecting the important role in different protein networks. Higher protein MCC and edge betweenness values indicate significant topological relevance within the biological network system.

At the transcriptional level, exposure of 24 h PAO1 biofilms to 5-FU resulted in a significant downregulation of type IV pili genes *pilA*, *pilI*, and *pilS*. Genes involved in flagellar motility such as *flhA*, *fliD*, and *motA* were downregulated, while *fliC* was upregulated at 100 µg/mL of 5-FU; *fleQ* was unchanged. The virulence-associated gene *lasB* was also downregulated. Treatment with 5-FU resulted in a significant downregulation of rhlAB, while the expression of rhlC remained largely unchanged (Fig 3). In 48 h PAO1 biofilms, treatment with 5-FU significantly upregulated selected DNA repair and stress-associated genes, including *nth, xthA*, *recJ*, *sbcB*, and *eddB* (Fig 3).

### Molecular Docking

**Fig 4:**
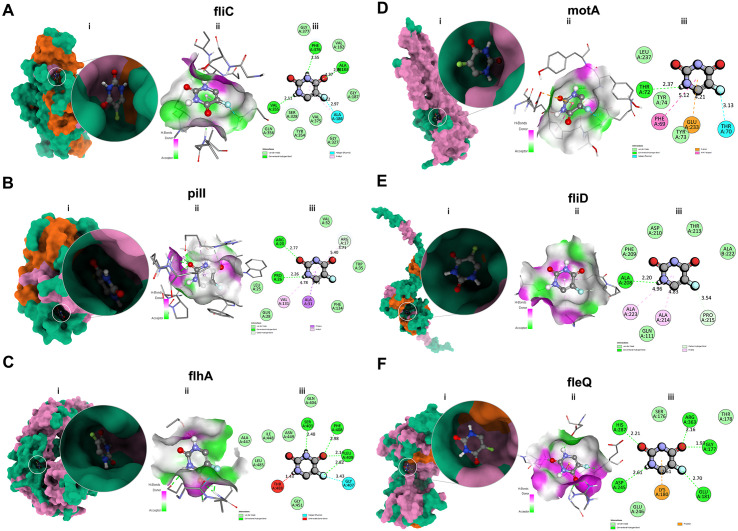
Molecular-docked complexes of (A) fliC, (B) pilI, (C) flhA, (D) motA, (E) fliD, (F) fleQ with 5-FU ligand. The left panel (i) shows the protein surface with predicted binding site in the white circle; pink represents the helices, orange represents the β strands, and green indicates loops. The middle panel (ii) shows the predicted hydrogen bonding surface interactions of the protein and ligand. The green dashed lines represent hydrogen bonds; blue dashed lines denote halogen interactions, and yellow dashed lines denote π-stacking interactions. Pink areas represent hydrogen bond donors, and green areas represent hydrogen acceptors. The right panel (iii) shows the interaction diagrams between 5-FU and the PAO1 target receptors.

Across all proteins, 5-FU showed predicted moderate and relatively uniform binding with docking energies ranging from −4.5 to −6.3 kcal/mol and hydrogen-bond interactions ranging from 1 to 6; and the detailed results are outlined in S3 Table. Moderate predicted binding scores were observed for pilI, fliC, and flhA of −4.9, −5.2, and −5.4 kcal/mol, each showing more than two hydrogen-bond interactions were observed, respectively; whereas the predicted weak binding affinity score for fleQ, motA, and fliD of −4.8, −4.5, and −4.5 kcal/mol were observed, respectively, showing minimum of 1 hydrogen-bond interaction. In addition, sbcB, recJ, and xthA predicted moderate affinity score of −5.6, −6.1, and −6.3 kcal/mol, respectively, and each showing more than 3 hydrogen-bond interactions. Moreover, the predicted contact residues for 5-FU, as shown in Fig. 4, include fliC (ALA183, PHE376, VAL355, and ALA186 ranging 2.0–2.84 Å distance); pilI (ARG20, PRO26, ARG17, ALA31, and VAL131 ranging 2.26–5.40 Å distance); flhA (LEU409, PHE408, SER403, GLY407, and THR488 ranging 1.43–3.43 Å distance); motA (ALA208, PRO215, ALA214, and ALA223 ranging 2.2–4.96 Å distance); fliD (THR72, THR70, GLU233, and PHE69 ranging 2.37–5.12 Å distance); and fleQ (ARG363, GLY177, GLU181, ASP245, HIS287, and LYS180 ranging 1.93–2.70 Å distance). Overall, 31 contacts involving 28 unique residues were identified and interaction distances ranging from 1.93 to 2.98 Å for hydrogen bond and from 1.43 to 5.98 Å for non-covalent hydrophobic/π interactions were predicted. These findings are based on in silico predictions and do not confirm direct protein inhibition, requiring further experimental validation (Fig 4).

## Discussion

Antimicrobial resistance remains a major global health challenge, and *P. aeruginosa* continues to pose a significant threat due to its intrinsic resistance mechanisms [56] and its ability to form persistent biofilms [57–59]. Biofilms protect bacterial cells from immune clearance and antimicrobial agents, thus enabling chronic and hard-to-treat infections [60,61]. Given the slow pace and high cost of developing new antibiotics, drug repurposing has emerged as a practical strategy for identifying anti-infective candidates with known pharmacological properties [18,62]. The anticancer drug 5-FU has shown antimicrobial and antibiofilm activity against both Gram-positive and Gram-negative organisms [17,19,26,63,64] and has also demonstrated synergy with gentamicin [26] and reversal of meropenem resistance [27]. Its antibacterial mechanisms have also been investigated in *Escherichia coli* using biochemical and transcriptomic analyses [65]. However, the molecular and phenotypic effects associated with 5-FU exposure on *P. aeruginosa* biofilm development have not been elucidated.

*Pseudomonas aeruginosa* virulence is organized within highly complex and multilayered regulatory networks that integrate numerous genes involved in motility, biofilm development, secretion systems, quorum sensing, and stress adaptation. Accordingly, virulence arises through coordinated changes across interconnected gene networks underscoring the integrated systems-level control of pathogenicity [66–68]. The molecular basis of 5-FU-mediated virulence modulation remains poorly defined, and this study examined its effects across key virulence-associated processes focusing on motility phenotypes, the expression of core motility genes, and the related virulence determinants that indirectly contribute to surface colonization. Given that 5-FU is a DNA-targeting pyrimidine analog, its impact on extracellular DNA production was evaluated at both early (24 h) and mature (48 h) biofilm stages alongside transcriptional changes in selected DNA repair-associated genes. The results provide an integrated framework to assess how DNA-targeting compounds influence virulence-related bacterial behavior.

Swimming, swarming, and twitching were significantly suppressed with 5-FU treatment consistent with downregulation of flagellar- and pili-associated genes. The increase in motility observed at the lowest 5-FU concentration suggests a non-linear hormetic response, where sub-inhibitory chemical stress may transiently enhance bacterial motility and virulence-associated behaviors through adaptive stress signaling pathways [69,70]. Consistent with the motility inhibition, *motA*, a core component of the MotA/MotB stator complex that generates flagellar torque and contributes to surface sensing and early biofilm initiation, was downregulated [71–73]. In parallel, reduced *fliD* expression, which is essential for FliC polymerization and proper filament assembly, is known to compromise motility, adhesion, and biofilm establishment [74,75]. In this context, the observed upregulation of *fliC* may reflect compensatory transcriptional regulation within the flagellar regulatory hierarchy rather than functional restoration of filament assembly [76,77]. Consistent with the loss of twitching motility, downregulation of *pilA* and *pilI* indicates impaired type IV pili-associated dynamics; *pilA* encodes the major pilin subunit, and suppression of type IV pili has been shown to disrupt twitching motility, surface adhesion and alter early biofilm formation in *Pseudomonas aeruginosa* [8,78,79].

The suppression of *lasB* and *rhlAB* by 5-FU likely further contributes to the observed motility defects. In addition to encoding elastase, *lasB* has been implicated in surface-associated biofilm behaviors, and *lasB*-deficient mutants display reduced bacterial attachment and impaired microcolony development [80,81]. Additionally, pharmacological inhibition of *lasB* has been shown to reduce bacterial burden and improve outcomes in infection models particularly during early stages when motility-driven colonization is essential, thus supporting its role in functional virulence and motility [82,83]. Similarly, rhamnolipids are a key modulator of surface motility, particularly swarming, by reducing surface tension and enabling coordinated cell movement [84,85]. The observed downregulation of *rhlAB* with *rhlC* unchanged suggests a reduction in total rhamnolipid output because *rhlC* depends on *rhlAB*-derived mono-rhamnolipids to synthesize di-rhamnolipids [86,87].

5-FU is a pyrimidine analog known to interfere with nucleotide metabolism and DNA synthesis, and our findings suggest that this activity is associated with a time-dependent change in eDNA dynamics during biofilm development. At 24 h, a stage at which eDNA accumulation is typically enhanced, 5-FU treatment resulted in a significant reduction in eDNA levels relative to untreated control. In contrast, at 48 h, higher concentrations of 5-FU were associated with increased eDNA accumulation, coinciding with upregulation of selected DNA repair- and stress-associated genes. However, these findings do not demonstrate that DNA repair directly caused the increase in eDNA. Rather, the concurrent increase in eDNA and the upregulation of selected DNA repair-associated gene expression suggests a stress-related transcriptional response following prolonged 5-FU exposure, rather than confirming direct activation of a specific DNA repair or SOS pathway. In bacterial cells, fluoroquinolones have been reported to activate SOS-associated and subsequently induce DNA repair pathways in Gram-negative bacteria such as *Escherichia coli* and *Acinetobacter baumannii* [88,89].

In eukaryotic systems, 5-FU induces a DNA damage response through thymidylate synthase inhibition and nucleotide imbalance, resulting in replication stress and activation of DNA repair pathways [90,91]. Furthermore, some studies have demonstrated that SOS induction in Gram-negative bacteria has been linked to increased extracellular DNA release largely through enhanced cell lysis and stress-induced DNA extrusion [92,93]. Putting all this together, we hypothesize that the increased eDNA and DNA repair gene upregulation observed at 48 h may likely represent a secondary consequence of 5-FU-associated DNA damage response and genomic stress. This interpretation is further supported by previous observations of increased dead-cell populations under the same treatment conditions [26].

Across both time points, 5-FU exposure produced coordinated but non-uniform transcriptional changes in *P. aeruginosa* genes associated with motility and stress-related pathways. PPI analysis demonstrated that transcriptionally affected genes were not isolated but embedded within a connected network where a limited subset of nodes exhibited higher degrees, MCC, and edge betweenness. Molecular docking suggested that 5-FU can associate with multiple proteins represented in the network with comparable binding patterns rather than a single dominant target, thus supporting a multi-target mode of interaction. Taken together, the data support that 5-FU influences *P. aeruginosa* at the systems level by simultaneously modulating gene expression and engaging multiple network-connected proteins rather than acting through a narrowly defined molecular target.

**Fig 5:**
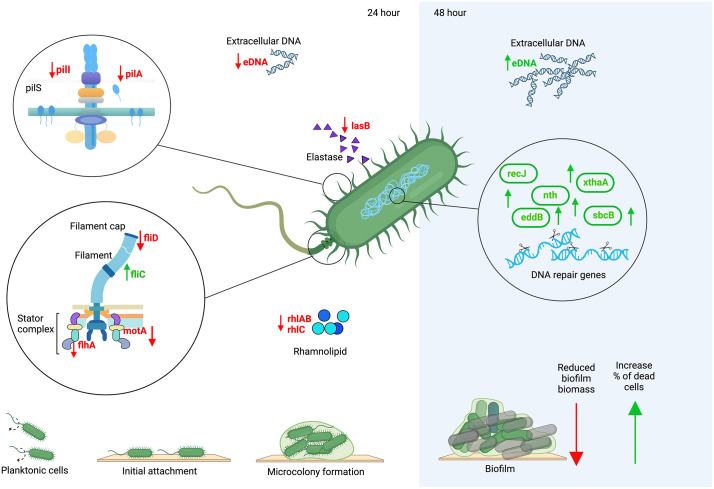
Proposed model summarizing the stage-dependent effects of 5-FU on *Pseudomonas aeruginosa* PAO1 biofilm development at 24 and 48 h. The schematic illustrates the impact of 5-FU on type IV pili, flagellar structures, rhamnolipid synthesis, LasB protease production, extracellular DNA (eDNA) levels, DNA repair genes, and biofilm-associated phenotypes. Elements displayed in red with downward arrows represent downregulated or reduced genes or phenotypes. Elements shown in green with upward arrows indicate upregulated or increased expression or phenotypes. Elements shown in black indicate no significant change. The left panel summarizes the molecular and phenotypic changes observed at 24 h, while the right panel shows the responses detected at 48 h. Created with BioRender.com.

Collectively, these findings integrating phenotypic and molecular data (Fig 5) indicate that 5-FU can impair motility, rhamnolipid-associated pathways, and reduce eDNA availability within the first 24 h of exposure. At 48 h, 5-FU is associated with increased eDNA accumulation and upregulation of DNA repair genes consistent with a stress-driven response. Together, these findings support a model in which 5-FU initially suppresses virulence-linked traits and biofilm formation as reported in previous studies [19,26,63], but with prolonged exposure may induce nucleotide stress responses that coincide with DNA damage that promotes upregulation of DNA repair genes and eDNA release. The increased eDNA levels observed at 48 h are most likely attributable to passive release resulting from cell lysis, consistent with our previous findings demonstrating increased cell death under 5-FU exposure [26]. Nevertheless, the potential contribution of active eDNA secretion by viable cells cannot be excluded and requires further experimental validation. Future studies using clinical isolates and in vivo models will help advance these findings toward translational relevance. In parallel, chemical optimization of 5-FU [25] or its combination with complementary agents may enhance antibacterial efficacy [26,27], in addition to its potential use as an early-stage adjunct therapy. Collectively, these observations highlight the potential of 5-FU as a candidate for repurposing in strategies aimed at combating multidrug-resistant infections.

This study has several limitations. Transcriptional data provide insight into regulatory responses but do not confirm functional outcomes for each gene; complementary proteomic or targeted genetic approaches would strengthen the mechanistic conclusions. Another limitation is the use of a single reference gene for RT-qPCR normalization. Although 16S rRNA is commonly used in *P. aeruginosa* gene expression studies, its stability under 5-FU exposure was not independently validated. Future studies should validate multiple reference genes under the same treatment conditions. In addition, the docking analysis provides theoretical insight and cannot establish direct protein interactions without experimental confirmation. Experiments were conducted on a single strain under defined laboratory conditions, which may not fully reflect clinical variability or represent the diversity of clinical isolates. The findings are limited to in vitro conditions, and in vivo studies are required to evaluate efficacy and potential toxicity within a complex biological environment before clinical translation. A further limitation is that the highest test concentration (100 µg/mL) may not be suitable for systemic clinical use due to potential host toxicity. Future work may investigate localized application, combination therapies, or chemical optimization of 5-FU to improve its antibacterial efficacy against *P. aeruginosa*. Additionally, long-term adaptation to 5-FU was beyond the scope of this work. Resistance development was not assessed; thus, the potential for 5-FU to induce resistance tolerance over prolonged exposure remains unclear. Future studies should examine whether prolonged exposure promotes tolerance, persistence, or resistance, and should define the dosing strategies and administration frequency required to maintain antibacterial efficacy. Despite these limitations, the findings of this study advance understanding of the phenotypic and transcriptional responses associated with the effects of 5-FU on *P. aeruginosa*, which may inform its translation toward clinical applications.

## Supporting information

S1 Fig Relative expression of 24 h *P. aeruginosa* biofilm-associated genes after treatment with increasing concentrations of 5-FU. Gene expression was quantified by quantitative real-time PCR, normalized to the 16S rRNA reference gene, and calculated using the 2^-ΔΔCt^ method. Values are expressed as fold change relative to the untreated control. Data represent mean ± SD from two independent experiments. Asterisks indicate significance levels: *p* < 0.05 (*), *p* < 0.01 (**), *p* < 0.001 (***), and *p* < 0.0001 (****).
(TIF)

S2 Fig Relative expression of 48 h *P. aeruginosa* biofilm-associated genes after treatment with increasing concentrations of 5-FU. Gene expression was quantified by quantitative real-time PCR, normalized to the 16S rRNA reference gene, and calculated using the 2^-ΔΔCt^ method. Values are expressed as fold change relative to the untreated control. Data represent mean ± SD from three independent experiments. Asterisks indicate significance levels: *p* < 0.05 (*), *p* < 0.01 (**), *p* < 0.001 (***), and *p* < 0.0001 (****).
(TIF)

S3 Fig Schematic diagram of PAO1 genes FC ratio treated with different 5-FU concentrations at 24 and 48 h exposure. Arrow depicts the direction and relative gene FC ratio for each regulation group. Green color indicates upregulation; and red indicates downregulation. Relative fold‑change ratios at 24 h showing upregulation of *fliC* and *rhlC*; and downregulation of *fleQ*, *pilS*, *flhA*, *lasB*, *motA*, *pilA*, *rhlAB*, *pilI*, and *pilD*. Relative FC ratios at 48 h showing upregulation of *nth, sbcB, xthA, recJ,* and *eddB.* The fixed height ratio of each regulations and genes were calculated and illustrated according to (1) fold regulation group ratio; and (2) the ratio of each gene FC value from the sum of FC on respective regulation group.
(TIF)

S4 Fig VINA docking results complex of 5-FU and PAO1 receptor. Molecular-docked complexes interaction of (A) lasB (B) xthA, (C) recJ, (D) pilS, (E) rhlR, (F)sbcB, (G) pilA, (H) rhlC, (I) eddB, (J) nth, with 5-FU ligand. The left panel (i) shows the protein surface with predicted binding site in the white circle; pink represents the helices, orange represents the β strands, and green indicates loops. The middle panel (ii) shows the hydrogen bonding surface interaction of protein and ligand. The green dashed lines represent hydrogen bonds; blue dash lines denote halogen interaction, and yellow dash lines denote pi-stacking interaction. Pink areas represent hydrogen bond donors, and green areas represent hydrogen acceptors. The right panel (iii) shows the interaction diagram of 5-FU and PAO1 receptors.
(TIF)

S1 Table PAO1 protein locus tags. (PDF)

S2 Table Receptor grid coordinates, box dimensions, and AutoDock VINA parameters. (PDF)

S3 Table Detailed molecular docking results obtained using AutoDock VINA. (PDF)

## References

[1] Qin S, Xiao W, Zhou C, Pu Q, Deng X, Lan L, et al. *Pseudomonas aeruginosa*: pathogenesis, virulence factors, antibiotic resistance, interaction with host, technology advances and emerging therapeutics. Signal Transduct Target Ther. 2022;7(1):199. doi: 10.1038/s41392-022-01056-1 35752612 PMC9233671

[2] Moradali MF, Ghods S, Rehm BHA. *Pseudomonas aeruginosa* Lifestyle: A Paradigm for Adaptation, Survival, and Persistence. Front Cell Infect Microbiol. 2017;7:39. doi: 10.3389/fcimb.2017.00039 28261568 PMC5310132

[3] Liao C, Huang X, Wang Q, Yao D, Lu W. Virulence factors of *Pseudomonas aeruginosa* and antivirulence strategies to combat its drug resistance. Front Cell Infect Microbiol. 2022;12:926758. doi: 10.3389/fcimb.2022.926758 35873152 PMC9299443

[4] Botelho J, Grosso F, Peixe L. Antibiotic resistance in *Pseudomonas aeruginosa* - Mechanisms, epidemiology and evolution. Drug Resist Updat. 2019;44:100640. doi: 10.1016/j.drup.2019.07.002 31492517

[5] Eid R, Dabar G, Hanna L-R, Saliba G, Riachy M, Choucair J, et al. Comparison of antimicrobial resistance in *Pseudomonas aeruginosa* from intensive care and non-intensive care units and its impact on treatment decisions. Sci Rep. 2025;15(1):11288. doi: 10.1038/s41598-025-90791-w 40175451 PMC11965343

[6] Centers for Disease Control and Prevention. Antibiotic Resistance Threats in the United States, 2019. Atlanta, GA: U.S. Department of Health and Human Services, CDC. 2019. https://www.cdc.gov/antimicrobial-resistance/media/pdfs/2019-ar-threats-report-508.pdf

[7] World Health Organization. WHO bacterial priority pathogens list, 2024: bacterial pathogens of public health importance to guide research, development and strategies to prevent and control antimicrobial resistance. Geneva: World Health Organization. 2024. https://www.who.int/publications/i/item/9789240093461

[8] Klausen M, Heydorn A, Ragas P, Lambertsen L, Aaes-Jørgensen A, Molin S, et al. Biofilm formation by *Pseudomonas aeruginosa* wild type, flagella and type IV pili mutants. Mol Microbiol. 2003;48(6):1511–24. doi: 10.1046/j.1365-2958.2003.03525.x 12791135

[9] Sauer K, Stoodley P, Goeres DM, Hall-Stoodley L, Burmølle M, Stewart PS, et al. The biofilm life cycle: expanding the conceptual model of biofilm formation. Nat Rev Microbiol. 2022;20(10):608–20. doi: 10.1038/s41579-022-00767-0 35922483 PMC9841534

[10] Matilla MA, Krell T. Targeting motility and chemotaxis as a strategy to combat bacterial pathogens. Microb Biotechnol. 2023;16(12):2205–11. doi: 10.1111/1751-7915.14306 37387327 PMC10686171

[11] Lynch MJ, Kurniyati K, Deshpande M, Charon NW, Li C, Crane BR. Inhibitors of Lysinoalanine Cross-Linking in the Flagella Hook as Antimicrobials against Spirochetes. ACS Chem Biol. 2025;20(3):620–31. doi: 10.1021/acschembio.4c00749 40000236 PMC12577791

[12] Campoccia D, Montanaro L, Arciola CR. Extracellular DNA (eDNA). A major ubiquitous element of the bacterial biofilm architecture. Int J Mol Sci. 2021;22:9100. doi: 10.3390/ijms22169100 34445806 PMC8396552

[13] Ferguson DL, Gloag ES, Parsek MR, Wozniak DJ. Extracellular DNA enhances biofilm integrity and mechanical properties of mucoid *Pseudomonas aeruginosa*. J Bacteriol. 2023;205(10):e0023823. doi: 10.1128/jb.00238-23 37791754 PMC10601617

[14] Sharma DK, Rajpurohit YS. Multitasking functions of bacterial extracellular DNA in biofilms. J Bacteriol. 2024;206(4):e0000624. doi: 10.1128/jb.00006-24 38445859 PMC11025335

[15] Thi MTT, Wibowo D, Rehm BHA. *Pseudomonas aeruginosa* Biofilms. Int J Mol Sci. 2020;21(22):8671. doi: 10.3390/ijms21228671 33212950 PMC7698413

[16] Imperi F, Massai F, Facchini M, Frangipani E, Visaggio D, Leoni L, et al. Repurposing the antimycotic drug flucytosine for suppression of *Pseudomonas aeruginosa* pathogenicity. Proc Natl Acad Sci U S A. 2013;110(18):7458–63. doi: 10.1073/pnas.1222706110 23569238 PMC3645532

[17] Farha MA, Brown ED. Drug repurposing for antimicrobial discovery. Nat Microbiol. 2019;4(4):565–77. doi: 10.1038/s41564-019-0357-1 30833727

[18] Barbarossa A, Rosato A, Corbo F, Clodoveo ML, Fracchiolla G, Carrieri A, et al. Non-Antibiotic Drug Repositioning as an Alternative Antimicrobial Approach. Antibiotics (Basel). 2022;11(6):816. doi: 10.3390/antibiotics11060816 35740222 PMC9220406

[19] Miró-Canturri A, Ayerbe-Algaba R, Smani Y. Drug repurposing for the treatment of bacterial and fungal infections. Front Microbiol. 2019;10:41. doi: 10.3389/fmicb.2019.00041 30745898 PMC6360151

[20] Di Bonaventura G, Lupetti V, Di Giulio A, Muzzi M, Piccirilli A, Cariani L, et al. Repurposing high-throughput screening identifies unconventional drugs with antibacterial and antibiofilm activities against *Pseudomonas aeruginosa* under experimental conditions relevant to cystic fibrosis. Microbiol Spectr. 2023;11(4):e0035223. doi: 10.1128/spectrum.00352-23 37306577 PMC10433973

[21] García-Contreras R, Martínez-Vázquez M, Velázquez Guadarrama N, Villegas Pañeda AG, Hashimoto T, Maeda T, et al. Resistance to the quorum-quenching compounds brominated furanone C-30 and 5-fluorouracil in *Pseudomonas aeruginosa* clinical isolates. Pathog Dis. 2013;68(1):8–11. doi: 10.1111/2049-632X.12039 23620228

[22] Kirienko DR, Revtovich AV, Kirienko NV. A high-content, phenotypic screen identifies fluorouridine as an inhibitor of pyoverdine biosynthesis and *Pseudomonas aeruginosa* virulence. mSphere. 2016;1(4):e00217-16. doi: 10.1128/mSphere.00217-16 27579370 PMC4999921

[23] Ueda A, Attila C, Whiteley M, Wood TK. Uracil influences quorum sensing and biofilm formation in *Pseudomonas aeruginosa* and fluorouracil is an antagonist. Microb Biotechnol. 2009;2(1):62–74. doi: 10.1111/j.1751-7915.2008.00060.x 21261882 PMC3815422

[24] Carullo G, Di Bonaventura G, Rossi S, Lupetti V, Tudino V, Brogi S, et al. Development of quinazolinone derivatives as modulators of virulence factors of *Pseudomonas aeruginosa* cystic fibrosis strains. Molecules. 2023;28(18):6535. doi: 10.3390/molecules28186535 37764311 PMC10536951

[25] Patil M, Serhii K, Garzino F, Gobert Q, Giorgio S, Raimundo J-M, et al. Synthesis and antimicrobial testing of 5-fluorouracil derivatives. Arch Pharm (Weinheim). 2023;356(7):e2300103. doi: 10.1002/ardp.202300103 37199697

[26] Niazy AA, Alrashed MM, Lambarte RNA, Niazy AA. 5-Fluorouracil inhibits bacterial growth and reduces biofilm in addition to having synergetic effects with gentamicin against *Pseudomonas aeruginosa*. Microorganisms. 2024;12(11):2257. doi: 10.3390/microorganisms12112257 39597647 PMC11596706

[27] Zhang M, Yang S, Liu Y, Zou Z, Zhang Y, Tian Y, et al. Anticancer agent 5-fluorouracil reverses meropenem resistance in carbapenem-resistant Gram-negative pathogens. Int J Antimicrob Agents. 2024;64(5):107337. doi: 10.1016/j.ijantimicag.2024.107337 39293771

[28] Ornston LN, Stanier RY. The conversion of catechol and protocatechuate to beta-ketoadipate by *Pseudomonas putida*. J Biol Chem. 1966;241(16):3776–86. doi: 10.1016/s0021-9258(18)99839-x 5916391

[29] Galanti L, Lebitasy MP, Hecq J-D, Cadrobbi J, Vanbeckbergen D, Jamart J. Long-term stability of 5-Fluorouracil in 0.9% sodium chloride after freezing, microwave thawing, and refrigeration. Can J Hosp Pharm. 2009;62(1):34–8. doi: 10.4212/cjhp.v62i1.115 22478863 PMC2826915

[30] Ha D-G, Kuchma SL, O’Toole GA. Plate-based assay for swarming motility in *Pseudomonas aeruginosa*. Methods Mol Biol. 2014;1149:67–72. doi: 10.1007/978-1-4939-0473-0_8 24818898 PMC9006052

[31] Ha D-G, Kuchma SL, O’Toole GA. Plate-based assay for swimming motility in *Pseudomonas aeruginosa*. Methods Mol Biol. 2014;1149:59–65. doi: 10.1007/978-1-4939-0473-0_7 24818897 PMC9007281

[32] Turnbull L, Whitchurch CB. Motility assay: twitching motility. Methods Mol Biol. 2014;1149:73–86. doi: 10.1007/978-1-4939-0473-0_9 24818899

[33] O’Hara MT, Shimozono TM, Dye KJ, Harris D, Yang Z. Surface hydrophilicity promotes bacterial twitching motility. mSphere. 2024;9(9):e0039024. doi: 10.1128/msphere.00390-24 39194233 PMC11423576

[34] Niazy AA, Lambarte RNA, Alghamdi HS. *De novo* pyrimidine synthesis pathway inhibition reduces motility virulence of *Pseudomonas aeruginosa* despite complementation. Journal of King Saud University - Science. 2022;34(4):102040. doi: 10.1016/j.jksus.2022.102040

[35] Balyimez A. Characterization of the *Pseudomonas aeruginosa* MepA, a unique metalloendopeptidase whose gene is a part of the Vfr regulon. Texas Tech University. 2013. https://hdl.handle.net/2346/89005 10.1186/1471-2180-13-269 PMC4222646 24279383

[36] Ma Y, Liu Y, Bi Y, Han X, Jin Y, Xu H, et al. OsaR (PA0056) functions as a repressor of the gene *fleQ* encoding an important motility regulator in *Pseudomonas aeruginosa*. J Bacteriol. 2021;203(20):e0014521. doi: 10.1128/JB.00145-21 34339300 PMC8459766

[37] Jain R, Kazmierczak BI. A conservative amino acid mutation in the master regulator FleQ renders *Pseudomonas aeruginosa* aflagellate. PLoS One. 2014;9(5):e97439. doi: 10.1371/journal.pone.0097439 24827992 PMC4020848

[38] Hu J, Xia Y, Xiong Y, Li X, Su X. Inhibition of biofilm formation by the antisense peptide nucleic acids targeted at the *motA* gene in *Pseudomonas aeruginosa* PAO1 strain. World J Microbiol Biotechnol. 2011;27(9):1981–7. doi: 10.1007/s11274-011-0658-x

[39] Bogiel T, Depka D, Rzepka M, Mikucka A. Decoding genetic features and antimicrobial susceptibility of *Pseudomonas aeruginosa* strains isolated from bloodstream infections. Int J Mol Sci. 2022;23(16):9208. doi: 10.3390/ijms23169208 36012468 PMC9409454

[40] Zhao F, Shi R, Ma F, Han S, Zhang Y. Oxygen effects on rhamnolipids production by *Pseudomonas aeruginosa*. Microb Cell Fact. 2018;17(1):39. doi: 10.1186/s12934-018-0888-9 29523151 PMC5844106

[41] Gnanadhas DP, Elango M, Datey A, Chakravortty D. Chronic lung infection by *Pseudomonas aeruginosa* biofilm is cured by L-Methionine in combination with antibiotic therapy. Sci Rep. 2015;5:16043. doi: 10.1038/srep16043 26521707 PMC4629202

[42] Olszak T, Danis-Wlodarczyk K, Arabski M, Gula G, Maciejewska B, Wasik S, et al. *Pseudomonas aeruginosa* PA5oct jumbo phage impacts planktonic and biofilm population and reduces its host virulence. Viruses. 2019;11(12):1089. doi: 10.3390/v11121089 31771160 PMC6950013

[43] Schmittgen TD, Livak KJ. Analyzing real-time PCR data by the comparative C(T) method. Nat Protoc. 2008;3(6):1101–8. doi: 10.1038/nprot.2008.73 18546601

[44] Zatorska B, Groger M, Moser D, Diab-Elschahawi M, Lusignani LS, Presterl E. Does Extracellular DNA Production Vary in Staphylococcal Biofilms Isolated From Infected Implants versus Controls?. Clin Orthop Relat Res. 2017;475(8):2105–13. doi: 10.1007/s11999-017-5266-0 28194715 PMC5498371

[45] Szklarczyk D, Nastou K, Koutrouli M, Kirsch R, Mehryary F, Hachilif R, et al. The STRING database in 2025: protein networks with directionality of regulation. Nucleic Acids Res. 2025;53(D1):D730–7. doi: 10.1093/nar/gkae1113 39558183 PMC11701646

[46] Shannon P, Markiel A, Ozier O, Baliga NS, Wang JT, Ramage D, et al. Cytoscape: a software environment for integrated models of biomolecular interaction networks. Genome Res. 2003;13(11):2498–504. doi: 10.1101/gr.1239303 14597658 PMC403769

[47] Berman HM, Westbrook J, Feng Z, Gilliland G, Bhat TN, Weissig H, et al. The Protein Data Bank. Nucleic Acids Res. 2000;28(1):235–42. doi: 10.1093/nar/28.1.235 10592235 PMC102472

[48] Roy A, Kucukural A, Zhang Y. I-TASSER: a unified platform for automated protein structure and function prediction. Nat Protoc. 2010;5(4):725–38. doi: 10.1038/nprot.2010.5 20360767 PMC2849174

[49] Winsor GL, Griffiths EJ, Lo R, Dhillon BK, Shay JA, Brinkman FSL. Enhanced annotations and features for comparing thousands of *Pseudomonas* genomes in the *Pseudomonas* genome database. Nucleic Acids Res. 2016;44(D1):D646-53. doi: 10.1093/nar/gkv1227 26578582 PMC4702867

[50] Nnyigide OS, Nnyigide TO, Lee S-G, Hyun K. Protein Repair and Analysis Server: A Web Server to Repair PDB Structures, Add Missing Heavy Atoms and Hydrogen Atoms, and Assign Secondary Structures by Amide Interactions. J Chem Inf Model. 2022;62(17):4232–46. doi: 10.1021/acs.jcim.2c00571 36000562

[51] Forli S, Huey R, Pique ME, Sanner MF, Goodsell DS, Olson AJ. Computational protein-ligand docking and virtual drug screening with the AutoDock suite. Nat Protoc. 2016;11(5):905–19. doi: 10.1038/nprot.2016.051 27077332 PMC4868550

[52] Kim S, Chen J, Cheng T, Gindulyte A, He J, He S, et al. PubChem 2019 update: improved access to chemical data. Nucleic Acids Res. 2019;47(D1):D1102–9. doi: 10.1093/nar/gky1033 30371825 PMC6324075

[53] Hanwell MD, Curtis DE, Lonie DC, Vandermeersch T, Zurek E, Hutchison GR. Avogadro: an advanced semantic chemical editor, visualization, and analysis platform. J Cheminform. 2012;4(1):17. doi: 10.1186/1758-2946-4-17 22889332 PMC3542060

[54] Trott O, Olson AJ. AutoDock Vina: improving the speed and accuracy of docking with a new scoring function, efficient optimization, and multithreading. J Comput Chem. 2010;31(2):455–61. doi: 10.1002/jcc.21334 19499576 PMC3041641

[55] Dassault Systèmes B. Discovery Studio 2024 Client. San Diego, CA: Dassault Systèmes. 2024.

[56] Pang Z, Raudonis R, Glick BR, Lin T-J, Cheng Z. Antibiotic resistance in *Pseudomonas aeruginosa*: mechanisms and alternative therapeutic strategies. Biotechnol Adv. 2019;37(1):177–92. doi: 10.1016/j.biotechadv.2018.11.013 30500353

[57] Yin R, Cheng J, Wang J, Li P, Lin J. Treatment of *Pseudomonas aeruginosa* infectious biofilms: challenges and strategies. Front Microbiol. 2022;13:955286. doi: 10.3389/fmicb.2022.955286 36090087 PMC9459144

[58] Macias-Valcayo A, Aguilera-Correa J-J, Broncano A, Parron R, Auñon A, Garcia-Cañete J, et al. Comparative In Vitro Study of Biofilm Formation and Antimicrobial Susceptibility in Gram-Negative Bacilli Isolated from Prosthetic Joint Infections. Microbiol Spectr. 2022;10(4):e0085122. doi: 10.1128/spectrum.00851-22 35876589 PMC9430931

[59] Hindieh P, Yaghi J, Assaf JC, Chokr A, Atoui A, Tzenios N, et al. Emerging Multimodal Strategies for Bacterial Biofilm Eradication: A Comprehensive Review. Microorganisms. 2025;13(12):2796. doi: 10.3390/microorganisms13122796 41471999 PMC12735920

[60] Koo H, Allan RN, Howlin RP, Stoodley P, Hall-Stoodley L. Targeting microbial biofilms: current and prospective therapeutic strategies. Nat Rev Microbiol. 2017;15(12):740–55. doi: 10.1038/nrmicro.2017.99 28944770 PMC5685531

[61] Jamal M, Ahmad W, Andleeb S, Jalil F, Imran M, Nawaz MA, et al. Bacterial biofilm and associated infections. J Chin Med Assoc. 2018;81(1):7–11. doi: 10.1016/j.jcma.2017.07.012 29042186

[62] Niazy AA, Alrashed MM, Niazy AA. Effect of 5-fluorouracil on *Pseudomonas aeruginosa*: impact on virulence, biofilm formation, and bacterial growth. Front Microbiol. 2025;16:1584479. doi: 10.3389/fmicb.2025.1584479 40756206 PMC12313690

[63] Sedlmayer F, Woischnig A-K, Unterreiner V, Fuchs F, Baeschlin D, Khanna N, et al. 5-Fluorouracil blocks quorum-sensing of biofilm-embedded methicillin-resistant *Staphylococcus aureus* in mice. Nucleic Acids Res. 2021;49(13):e73. doi: 10.1093/nar/gkab251 33856484 PMC8287944

[64] Diógenes EM, Mesquita FP, da Silva EL, Pereira VC, de Souza PRH, Dos Santos PVC, et al. Effect of 5-fluorouracil on *Escherichia coli* and *Enterococcus* spp.: insights into the selective pressures caused by this cytotoxic drug. Microb Pathog. 2025;206:107701. doi: 10.1016/j.micpath.2025.107701 40368067

[65] Zhang M, Song H, Yang S, Zhang Y, Tian Y, Wang Y, et al. Deciphering the antibacterial mechanisms of 5-fluorouracil in *Escherichia coli* through biochemical and transcriptomic analyses. Antibiotics (Basel). 2024;13(6):528. doi: 10.3390/antibiotics13060528 38927194 PMC11200800

[66] Kanehisa M, Goto S. KEGG: kyoto encyclopedia of genes and genomes. Nucleic Acids Res. 2000;28(1):27–30. doi: 10.1093/nar/28.1.27 10592173 PMC102409

[67] Shao X, Yao C, Ding Y, Hu H, Qian G, He M, et al. The transcriptional regulators of virulence for *Pseudomonas aeruginosa*: therapeutic opportunity and preventive potential of its clinical infections. Genes Dis. 2022;10(5):2049–63. doi: 10.1016/j.gendis.2022.09.009 37492705 PMC10363592

[68] Huang J, Sun Y, Chen F, Li S, You X, Han L. Global transcription factors analyses reveal hierarchy and synergism of regulatory networks and master virulence regulators in *Pseudomonas aeruginosa*. eLife. 2026;14:RP103346. doi: 10.7554/eLife.103346.4 PMC13086498 41989285

[69] Iavicoli I, Fontana L, Agathokleous E, Santocono C, Russo F, Vetrani I, et al. Hormetic dose responses induced by antibiotics in bacteria: A phantom menace to be thoroughly evaluated to address the environmental risk and tackle the antibiotic resistance phenomenon. Sci Total Environ. 2021;798:149255. doi: 10.1016/j.scitotenv.2021.149255 34340082

[70] Linares JF, Gustafsson I, Baquero F, Martinez JL. Antibiotics as intermicrobial signaling agents instead of weapons. Proc Natl Acad Sci U S A. 2006;103(51):19484–9. doi: 10.1073/pnas.0608949103 17148599 PMC1682013

[71] Schniederberend M, Williams JF, Shine E, Shen C, Jain R, Emonet T, et al. Modulation of flagellar rotation in surface-attached bacteria: A pathway for rapid surface-sensing after flagellar attachment. PLoS Pathog. 2019;15(11):e1008149. doi: 10.1371/journal.ppat.1008149 31682637 PMC6855561

[72] Sánchez-Jiménez A, Llamas MA, Marcos-Torres FJ. Transcriptional Regulators Controlling Virulence in *Pseudomonas aeruginosa*. Int J Mol Sci. 2023;24(15):11895. doi: 10.3390/ijms241511895 37569271 PMC10418997

[73] Zheng X, Gomez-Rivas EJ, Lamont SI, Daneshjoo K, Shieh A, Wozniak DJ, et al. The surface interface and swimming motility influence surface-sensing responses in *Pseudomonas aeruginosa*. Proc Natl Acad Sci U S A. 2024;121(39):e2411981121. doi: 10.1073/pnas.2411981121 39284057 PMC11441478

[74] Postel S, Deredge D, Bonsor DA, Yu X, Diederichs K, Helmsing S, et al. Bacterial flagellar capping proteins adopt diverse oligomeric states. Elife. 2016;5:e18857. doi: 10.7554/eLife.18857 27664419 PMC5072837

[75] Bouteiller M, Dupont C, Bourigault Y, Latour X, Barbey C, Konto-Ghiorghi Y, et al. *Pseudomonas* Flagella: Generalities and Specificities. Int J Mol Sci. 2021;22(7):3337. doi: 10.3390/ijms22073337 33805191 PMC8036289

[76] Wolfgang MC, Jyot J, Goodman AL, Ramphal R, Lory S. *Pseudomonas aeruginosa* regulates flagellin expression as part of a global response to airway fluid from cystic fibrosis patients. Proc Natl Acad Sci U S A. 2004;101(17):6664–8. doi: 10.1073/pnas.0307553101 15084751 PMC404102

[77] Dasgupta N, Wolfgang MC, Goodman AL, Arora SK, Jyot J, Lory S, et al. A four-tiered transcriptional regulatory circuit controls flagellar biogenesis in *Pseudomonas aeruginosa*. Mol Microbiol. 2003;50(3):809–24. doi: 10.1046/j.1365-2958.2003.03740.x 14617143

[78] Marko VA, Kilmury SLN, MacNeil LT, Burrows LL. *Pseudomonas aeruginosa* type IV minor pilins and PilY1 regulate virulence by modulating FimS-AlgR activity. PLoS Pathog. 2018;14(5):e1007074. doi: 10.1371/journal.ppat.1007074 29775484 PMC5979040

[79] Rossy T, Distler T, Meirelles LA, Pezoldt J, Kim J, Talà L, et al. *Pseudomonas aeruginosa* type IV pili actively induce mucus contraction to form biofilms in tissue-engineered human airways. PLoS Biol. 2023;21(8):e3002209. doi: 10.1371/journal.pbio.3002209 37527210 PMC10393179

[80] Yu H, He X, Xie W, Xiong J, Sheng H, Guo S, et al. Elastase LasB of *Pseudomonas aeruginosa* promotes biofilm formation partly through rhamnolipid-mediated regulation. Can J Microbiol. 2014;60(4):227–35. doi: 10.1139/cjm-2013-0667 24693981

[81] Tielen P, Rosenau F, Wilhelm S, Jaeger K-E, Flemming H-C, Wingender J. Extracellular enzymes affect biofilm formation of mucoid *Pseudomonas aeruginosa*. Microbiology (Reading). 2010;156(Pt 7):2239–52. doi: 10.1099/mic.0.037036-0 20360178

[82] Galdino ACM, Viganor L, de Castro AA, da Cunha EFF, Mello TP, Mattos LM, et al. Disarming *Pseudomonas aeruginosa* virulence by the inhibitory action of 1,10-phenanthroline-5,6-dione-based compounds: elastase B (LasB) as a chemotherapeutic target. Front Microbiol. 2019;10:1701. doi: 10.3389/fmicb.2019.01701 31428062 PMC6688126

[83] Konstantinović J, Kany AM, Alhayek A, Abdelsamie AS, Sikandar A, Voos K, et al. Inhibitors of the elastase LasB for the treatment of *Pseudomonas aeruginosa* lung infections. ACS Cent Sci. 2023;9(12):2205–15. doi: 10.1021/acscentsci.3c01102 38161367 PMC10755728

[84] Caiazza NC, Shanks RMQ, O’Toole GA. Rhamnolipids modulate swarming motility patterns of *Pseudomonas aeruginosa*. J Bacteriol. 2005;187(21):7351–61. doi: 10.1128/JB.187.21.7351-7361.2005 16237018 PMC1273001

[85] O’Loughlin CT, Miller LC, Siryaporn A, Drescher K, Semmelhack MF, Bassler BL. A quorum-sensing inhibitor blocks *Pseudomonas aeruginosa* virulence and biofilm formation. Proc Natl Acad Sci U S A. 2013;110(44):17981–6. doi: 10.1073/pnas.1316981110 24143808 PMC3816427

[86] Van Gennip M, Christensen LD, Alhede M, Phipps R, Jensen PØ, Christophersen L, et al. Inactivation of the rhlA gene in *Pseudomonas aeruginosa* prevents rhamnolipid production, disabling the protection against polymorphonuclear leukocytes. APMIS. 2009;117(7):537–46. doi: 10.1111/j.1600-0463.2009.02466.x 19594494 PMC2997331

[87] Sharma J, Sundar D, Srivastava P. Biosurfactants: Potential Agents for Controlling Cellular Communication, Motility, and Antagonism. Front Mol Biosci. 2021;8:727070. doi: 10.3389/fmolb.2021.727070 34708073 PMC8542798

[88] Dörr T, Lewis K, Vulić M. SOS response induces persistence to fluoroquinolones in *Escherichia coli*. PLoS Genet. 2009;5(12):e1000760. doi: 10.1371/journal.pgen.1000760 20011100 PMC2780357

[89] Geisinger E, Vargas-Cuebas G, Mortman NJ, Syal S, Dai Y, Wainwright EL, et al. The Landscape of Phenotypic and Transcriptional Responses to Ciprofloxacin in *Acinetobacter baumannii*: Acquired Resistance Alleles Modulate Drug-Induced SOS Response and Prophage Replication. mBio. 2019;10(3):e01127-19. doi: 10.1128/mBio.01127-19 31186328 PMC6561030

[90] Longley DB, Harkin DP, Johnston PG. 5-fluorouracil: mechanisms of action and clinical strategies. Nat Rev Cancer. 2003;3(5):330–8. doi: 10.1038/nrc1074 12724731

[91] Kunz C, Focke F, Saito Y, Schuermann D, Lettieri T, Selfridge J, et al. Base excision by thymine DNA glycosylase mediates DNA-directed cytotoxicity of 5-fluorouracil. PLoS Biol. 2009;7(4):e91. doi: 10.1371/journal.pbio.1000091 19402749 PMC2671560

[92] Demjanenko P, Zheng S, Crane JK. SOS-Inducing Drugs Trigger Nucleic Acid Release and Biofilm Formation in Gram-Negative Bacteria. Biomolecules. 2024;14(3):321. doi: 10.3390/biom14030321 38540741 PMC10967838

[93] Crane JK, Yang T. Rapid assembly of biofilms from DNA released by SOS-inducing drugs in enteric bacteria. Sci Rep. 2025;15(1):12711. doi: 10.1038/s41598-025-96943-2 40223123 PMC11994792

