# Supplementary figures and images for: 5-Fluorouracil modulates motility and biofilm-associated gene expression in Pseudomonas aeruginosa
Source: PLoS One. 2026 Jul 23;21(7):e0354473. doi: 10.1371/journal.pone.0354473 (PMC13395363; doi:10.1371/journal.pone.0354473)

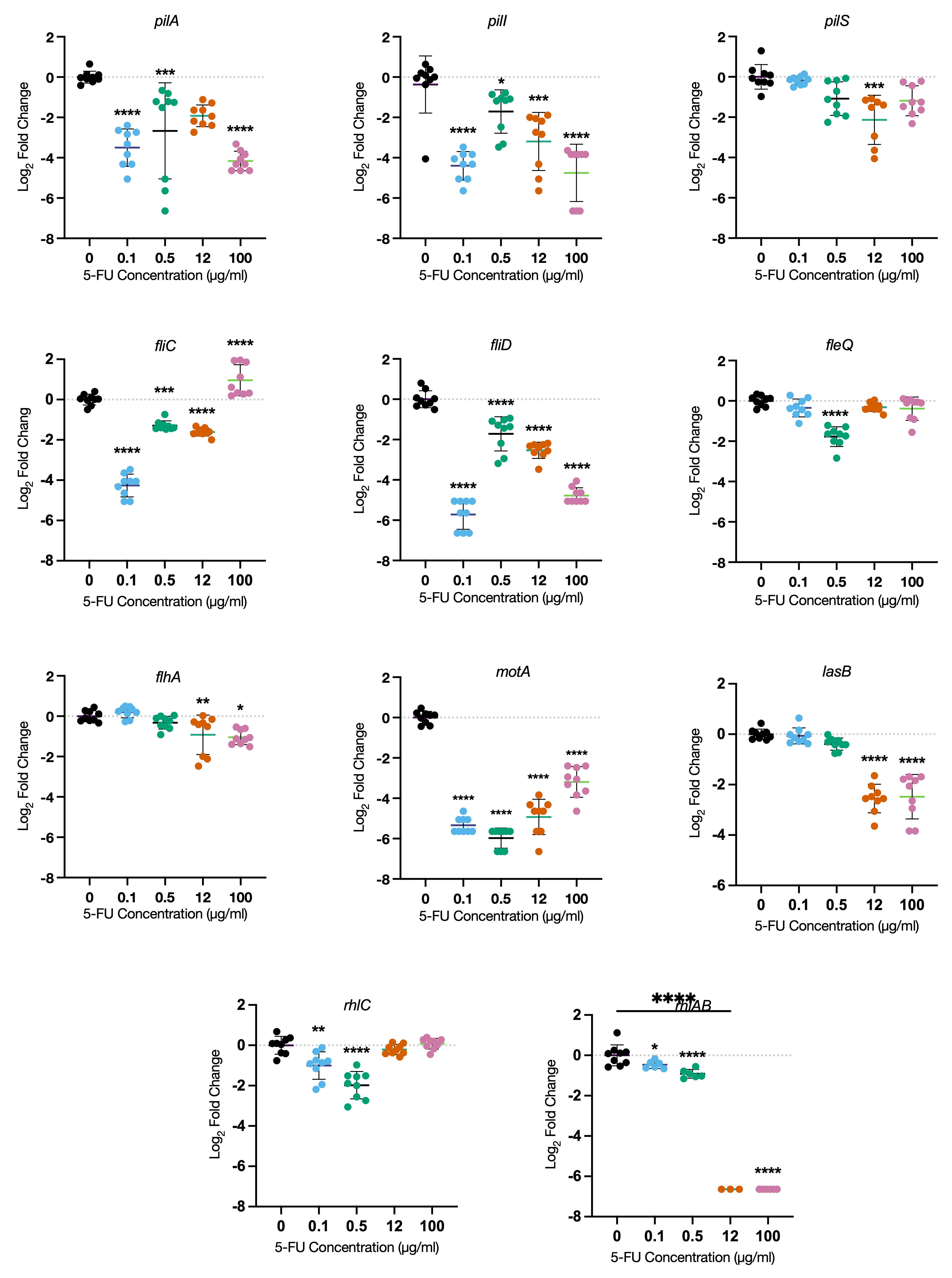

Supplement: S1 Fig — Gene expression was quantified by quantitative real-time PCR, normalized to the 16S rRNA reference gene, and calculated using the 2-ΔΔCt method. Values are expressed as fold change relative to the untreated control. Data represent mean ± SD from two independent experiments. Asterisks indicate significance levels: p < 0.05 (*), p < 0.01 (**), p < 0.001 (***), and p < 0.0001 (****). (TIF) [file pone.0354473.s001.tif]

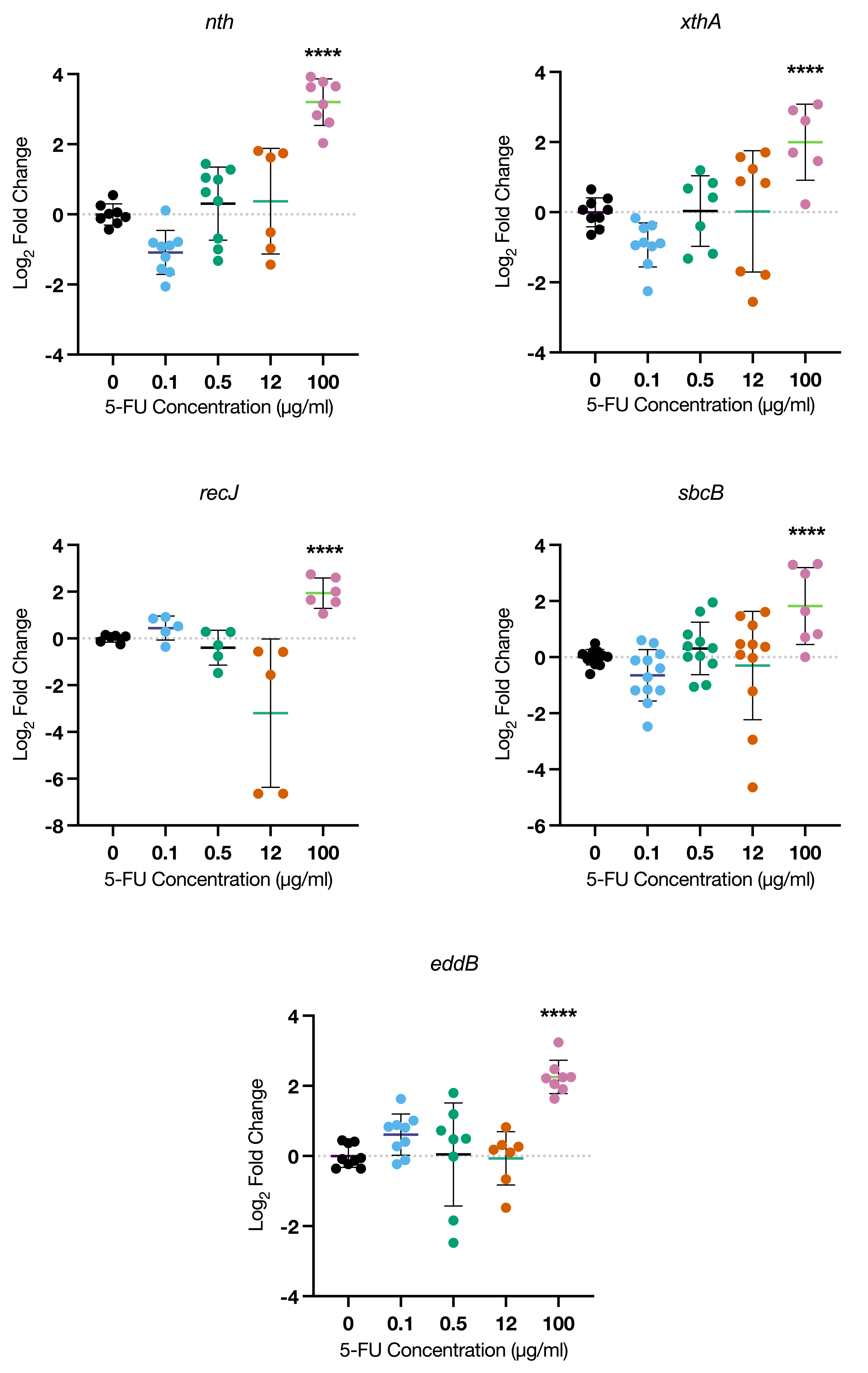

Supplement: S2 Fig — Gene expression was quantified by quantitative real-time PCR, normalized to the 16S rRNA reference gene, and calculated using the 2-ΔΔCt method. Values are expressed as fold change relative to the untreated control. Data represent mean ± SD from three independent experiments. Asterisks indicate significance levels: p < 0.05 (*), p < 0.01 (**), p < 0.001 (***), and p < 0.0001 (****). (TIF) [file pone.0354473.s002.tif]

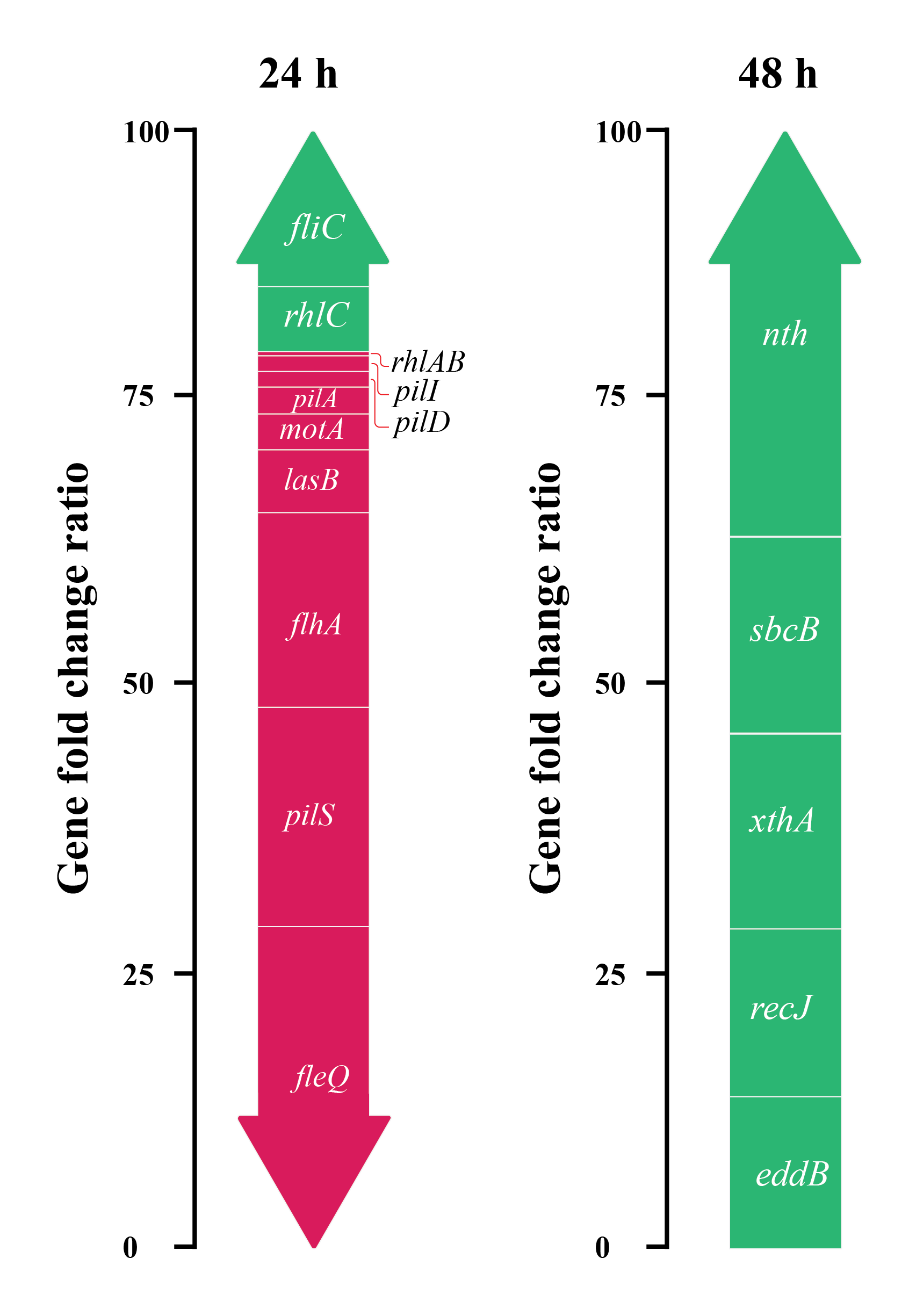

Supplement: S3 Fig — Arrow depicts the direction and relative gene FC ratio for each regulation group. Green color indicates upregulation; and red indicates downregulation. Relative fold‑change ratios at 24 h showing upregulation of fliC and rhlC; and downregulation of fleQ, pilS, flhA, lasB, motA, pilA, rhlAB, pilI, and pilD. Relative FC ratios at 48 h showing upregulation of nth, sbcB, xthA, recJ, and eddB. The fixed height ratio of each regulations and genes were calculated and illustrated according to (1) fold regulation group ratio; and (2) the ratio of each gene FC value from the sum of FC on respective regulation group. (TIF) [file pone.0354473.s003.tif]

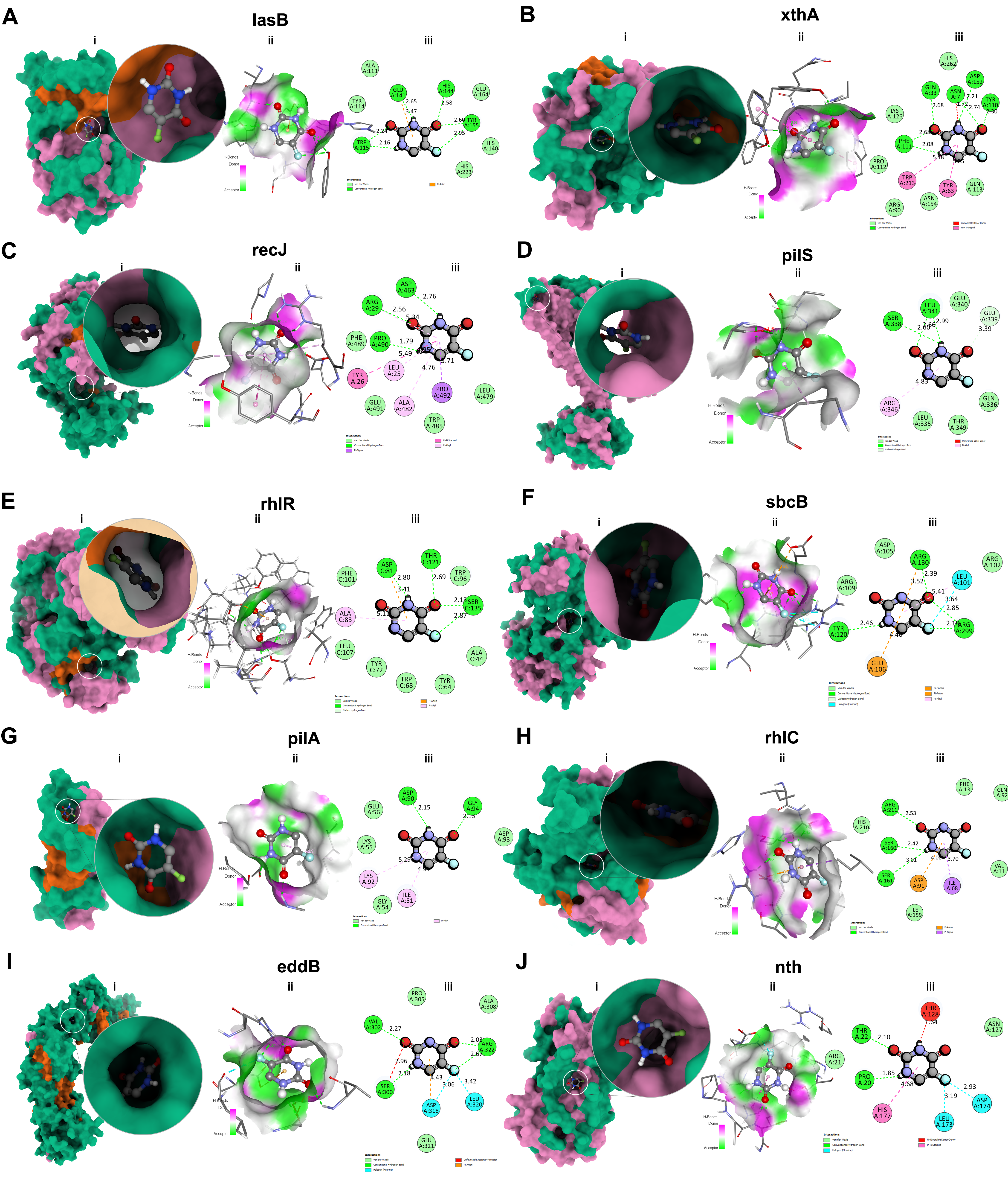

Supplement: S4 Fig — Molecular-docked complexes interaction of (A) lasB (B) xthA, (C) recJ, (D) pilS, (E) rhlR, (F)sbcB, (G) pilA, (H) rhlC, (I) eddB, (J) nth, with 5-FU ligand. The left panel (i) shows the protein surface with predicted binding site in the white circle; pink represents the helices, orange represents the β strands, and green indicates loops. The middle panel (ii) shows the hydrogen bonding surface interaction of protein and ligand. The green dashed lines represent hydrogen bonds; blue dash lines denote halogen interaction, and yellow dash lines denote pi-stacking interaction. Pink areas represent hydrogen bond donors, and green areas represent hydrogen acceptors. The right panel (iii) shows the interaction diagram of 5-FU and PAO1 receptors. (TIF) [file pone.0354473.s004.tif]
