# Supplementary material for: 5-Fluorouracil modulates motility and biofilm-associated gene expression in Pseudomonas aeruginosa
Source: PLoS One. 2026 Jul 23;21(7):e0354473. doi: 10.1371/journal.pone.0354473 (PMC13395363; doi:10.1371/journal.pone.0354473)
Supplement: S1 Table — (PDF) [file pone.0354473.s005.pdf]

**S1 Table. PAO1 protein locus tags.**

| PAO1 protein | Locus tag | Description |
|---|---|---|
| eddB | PA3909 | Extracelullar DNA degradation protein, EddB |
| fleQ | PA1097 | Transcriptional regulator FleQ |
| flhA | PA1452 | Flagellar biosynthesis protein FlhA |
| fliC | PA1092 | Flagellin type B |
| fliD | PA1094 | Flagellar capping protein FliD |
| lasB | PA3724 | Elastase LasB |
| motA | PA4954 | Chemotaxis protein MotA |
| nth | PA3495 | Endonuclease III |
| pilA | PA4525 | Type 4 fimbrial precursor PilA |
| pilI | PA0410 | Twitching motility protein PilI |
| pilS | PA4546 | Two-component sensor PilS |
| recJ | PA3725 | Single-stranded-DNA-specific exonuclease RecJ |
| rhlC | PA1130 | Rhamnosyltransferase 2 |
| rhlR | PA3477 | Transcriptional regulator RhlR |
| sbcB | PA4316 | Exodeoxyribonuclease I |
| xthA | PA2545 | Exodeoxyribonuclease III |
