# Supplementary material for: 5-Fluorouracil modulates motility and biofilm-associated gene expression in Pseudomonas aeruginosa
Source: PLoS One. 2026 Jul 23;21(7):e0354473. doi: 10.1371/journal.pone.0354473 (PMC13395363; doi:10.1371/journal.pone.0354473)
Supplement: S2 Table — (PDF) [file pone.0354473.s006.pdf]

S2 Table. Receptor grid coordinates, box dimensions, and AutoDock VINA parameters.

PAO1 Receptor	Box XYZ coordinates	Set box size XYZ (Å)	Exhaust-iveness	Modes	Energy range	Grid space
eddB	x: -0.818<br>y: -0.197<br>z: 0.008	126 × 126 ×126	128	40	15	1.0
fleQ	x: 28.602<br>y: 13.563<br>z: 0.143	126 × 126 ×126	128	40	15	0.5
flhA	x: 28.602<br>y: 13.563<br>z: 0.143	126 × 126 ×126	128	40	15	0.7
fliC	x: -32.949<br>y: -2.792<br>z: 21.913	126 × 126 ×126	128	40	15	0.6
fliD	x: 58.094<br>y: 145.564<br>z: 55.914	126 × 126 ×126	128	40	15	1.0
lasB	x: 0.128<br>y: -3.288<br>z: 10.315	126 × 126 ×126	128	40	15	0.4
motA	x: 78.309<br>y: 78.147<br>z: 78.896	126 × 126 ×126	128	40	15	0.6
nth	x: 0.203<br>y: 0.688<br>z: 0.046	126 × 126 ×126	128	40	15	0.5
pilA	x: 6.846<br>y: 0.884<br>z: 27.057	126 × 126 ×126	128	40	15	0.4
pilI	x: 55.61<br>y: 55.683<br>z: 55.32	126 × 126 ×126	128	40	15	0.4
pilS	x: 83.852<br>y: 83.007<br>z: 83.449	126 × 126 ×126	128	40	15	0.7
recJ	x: 0.238<br>y: 0.238<br>z: -0.654	126 × 126 ×126	128	40	15	0.6
rhlC	x: 63.029<br>y: 62.72<br>z: 63.091	126 × 126 ×126	128	40	15	0.5
rhlR	x: 106.681<br>y: 115.143<br>z: 136.512	126 × 126 ×126	128	40	15	0.5
sbcB	x: 0.053<br>y: 0.448<br>z: -0.343	126 × 126 ×126	128	40	15	0.6

xthA	x: 0.452<br>y: -0.341<br>z: -0.075	126 ×126 ×126	128	40	15	0.4
