# Supplementary material for: 5-Fluorouracil modulates motility and biofilm-associated gene expression in Pseudomonas aeruginosa
Source: PLoS One. 2026 Jul 23;21(7):e0354473. doi: 10.1371/journal.pone.0354473 (PMC13395363; doi:10.1371/journal.pone.0354473)
Supplement: S3 Table — (PDF) [file pone.0354473.s007.pdf]

S3 Table. Detailed molecular docking results obtained using AutoDock VINA.

5-FU-receptor complex	Affinity (kcal/mol)	Total H-bonds	Residue – Length (Interaction)
eddB	–5.2	4	ARG322 – 2.03 (H-Bond)<br>ARG322 – 2.67 (H-Bond)<br>SER302 – 2.27 (H-Bond)<br>VAL302 – 2.27 (H-Bond)<br>ASP318 – 4.43 (Pi-Anion)<br>ASP318 – 3.42 (Halogen (Fluorine))<br>LEU320 – 3.42 (Halogen (Fluorine))<br>SER300 – 2.96 (Unfavorable Acceptor-Acceptor)<br><br>ALA308 – (vdW)<br>GLU321 – (vdW)<br>PRO305 – (vdW)
fleQ	–4.8	5	ARG363 – 2.16 (H-Bond)<br>GLY177 – 1.93 (H-Bond)<br>GLU181 – 2.70 (H-Bond)<br>ASP245 – 2.61 (H-Bond)<br>HIS287 – 2.21 (H-Bond)<br>LYS180– 2.61 (Pi-Cation)<br><br>GLU246 – (vdW)<br>SER176 – (vdW)<br>THR178 – (vdW)
flhA	–5.4	4	LEU409 – 2.14 (H-Bond)<br>LEU409 – 2.82 (H-Bond)<br>PHE408 – 2.98 (H-Bond)<br>SER403 – 2.48 (H-Bond)<br>GLY407 – 3.43 (Halogen (Fluorine))<br>THR488 – 1.43 (Unfavorable Donor-Donor)<br><br>ALA447 – (vdW)<br>ASN449 – (vdW)<br>GLN404 – (vdW)<br>GLY451 – (vdW)<br>ILE448 – (vdW)<br>LEU485 – (vdW)
fliC	–5.2	3	ALA183 – 2.0 (H-Bond)<br>PHE376 – 2.55 (H-Bond)<br>VAL355 – 2.11 (H-Bond)<br>ALA186– 2.84 (Halogen (Fluorine))<br>ALA186– 2.55 (Pi-Alkyl)<br><br>GLN356 – (vdW)<br>GLY187 – (vdW)<br>GLY327 – (vdW)<br>GLY377 – (vdW)

			TYR354 – (vdW)<br>SER328 – (vdW)<br>VAL182 – (vdW)<br>VAL375 – (vdW)
fliD	–4.5	1	THR72 – 2.37 (H-Bond)<br>THR70 – 3.13 (Halogen (Fluorine))<br>GLU233 – 4.21 (Pi-Anion)<br>PHE69 – 5.12 (Pi-Pi T-shaped)<br><br>LEU237– (vdW)<br>TYR73 – (vdW)<br>TYR74 – (vdW)
lasB	–4.9	6	GLU141 – 2.65 (H-Bond)<br>HIS144 – 2.58 (H-Bond)<br>TRP115 – 2.24 (H-Bond)<br>TRP115 – 2.16 (H-Bond)<br>TYR155 – 2.60 (H-Bond)<br>TYR155 – 2.95 (H-Bond)<br><br>ALA113 – (vdW)<br>GLU164 – (vdW)<br>HIS140 – (vdW)<br>HIS223 – (vdW)<br>TYR114 – (vdW)
motA	–4.5	1	ALA208 – 2.20 (H-Bond)<br>PRO215 – 2.20 (Carbon H-Bond)<br>ALA214 – 4.83 (Pi-Alkyl)<br>ALA223 – 4.96 (Pi-Alkyl)<br><br>ALA222 – (vdW)<br>ASP210 – (vdW)<br>GLN111 – (vdW)<br>PHE209 – (vdW)<br>PRO215– (vdW)
nth	–4.8	2	PRO20 – 1.85 (H-Bond)<br>THR22 – 2.10 (H-Bond)<br>LEU173 – 3.19 (Halogen (Fluorine))<br>ASP174 – 2.93 (Halogen (Fluorine))<br>HIS177 – 4.68 (Pi-Pi Stacked)<br>THR128 – 1.64 (Unfavorable Donor-Donor)<br><br>ARG21 – (vdW)<br>ASN127 – (vdW)
pilA	–4.6	2	ASP90 – 2.15 (H-Bond)<br>GLY94 – 2.13 (H-Bond)<br>LYS92 – 5.29 (Pi-Alkyl)<br>ILE51 – 4.99 (Pi-Alkyl)<br><br>ASP93 – (vdW)

			GLU56 – (vdW)<br>GLY54 – (vdW)<br>LYS55 – (vdW)
pilI	–4.9	2	ARG20 – 2.77 (H-Bond)<br>PRO26 – 2.26 (H-Bond)<br>ARG17 – 3.71 (Carbon H-Bond)<br>ALA31 – 3.71 (Pi-Sigma)<br>ARG17 – 5.40 (Pi-Alkyl)<br>VAL131 – 4.78 (Pi-Alkyl)<br><br>GLN28 – (vdW)<br>LEU25 – (vdW)<br>PHE134 – (vdW)<br>TRP35 – (vdW)<br>VAL32 – (vdW)
pilS	–4.7	3	LEU341 – 2.99 (H-Bond)<br>LEU341 – 2.66 (H-Bond)<br>SER338 – 2.60 (H-Bond)<br>GLU339 – 3.39 (Carbon H-Bond)<br>ARG346 – 4.83 (Pi-Alkyl)<br><br>GLN336 – (vdW)<br>GLU339 – (vdW)<br>GLU340 – (vdW)<br>LEU335 – (vdW)<br>THR349 – (vdW)
recJ	–6.1	3	ARG29 – 2.56 (H-Bond)<br>ASP463 – 2.76 (H-Bond)<br>PRO490 – 1.79 (H-Bond)<br>PRO492 – 3.71 (Pi-Sigma)<br>TYR26 – 5.49 (Pi-Pi Stacked)<br>ALA482 – 4.76 (Pi-Alkyl)<br>ARG29 – 5.34 (Pi-Alkyl)<br>LEU25 – 5.49 (Pi-Alkyl)<br><br>PHE489 – (vdW)<br>LEU479 – (vdW)<br>TRP485 – (vdW)<br>GLU491 – (vdW)
rhlC	–5.1	3	ARG211 – 2.53 (H-Bond)<br>SER160 – 2.42 (H-Bond)<br>SER161 – 3.01 (H-Bond)<br>ASP91 – 4.08 (Pi-Anion)<br>ILE68 – 3.70 (Pi-Sigma)<br><br>GLN92 – (vdW)<br>ILE159 – (vdW)<br>HIS210 – (vdW)<br>PHE13 – (vdW)<br>VAL11 – (vdW)

rhlR	–5.6	4	ASP81 – 2.80 (H-Bond)<br>SER135 – 2.13 (H-Bond)<br>SER135 – 2.87 (H-Bond)<br>THR121 – 2.69 (H-Bond)<br>ASP81 – 3.41 (Pi-Anion)<br>ALA83 – 5.11 (Pi-Alkyl)<br><br>ALA44 – (vdW)<br>LEU107 – (vdW)<br>TRP68 – (vdW)<br>TYR64 – (vdW)<br>TYR72 – (vdW)<br>PHE101 – (vdW)
sbcB	–5.6	4	ARG130 – 2.39 (H-Bond)<br>ARG299 – 2.85 (H-Bond)<br>ARG299 – 2.18 (H-Bond)<br>TYR120 – 2.46 (H-Bond)<br>LEU101 – 3.64 (Halogen (Fluorine))<br>ARG130 – 3.52 (Pi-Cation)<br>GLU106 – 4.40 (Pi-Cation)<br>LEU101 – 5.41 (Pi-Alkyl)<br><br>ARG109 – (vdW)<br>ASP105 – (vdW)<br>ARG102 – (vdW)
xthA	–6.3	6	ASN7 – 2.74 (H-Bond)<br>ASP152 – 2.21 (H-Bond)<br>GLN33 – 2.68 (H-Bond)<br>PHE111 – 2.68 (H-Bond)<br>PHE111 – 2.08 (H-Bond)<br>TYR110 – 2.30 (H-Bond)<br>TRP213 – 5.48 (Pi-Pi T-shaped)<br>TYR63 – 5.48 (Pi-Pi T-shaped)<br>ASN7 – 1.72 (Pi-Pi T-shaped)<br><br>ASN9 – (vdW)<br>ASN154– (vdW)<br>ARG90 – (vdW)<br>GLN113 – (vdW)<br>GLU34 – (vdW)<br>LYS126 – (vdW)<br>PRO112 – (vdW)
